# Functional Characterisation of ClpP Mutations Conferring Resistance to Acyldepsipeptide Antibiotics in Firmicutes

**DOI:** 10.1002/cbic.201900787

**Published:** 2020-04-09

**Authors:** Imran T. Malik, Rebeca Pereira, Marie‐Theres Vielberg, Christian Mayer, Jan Straetener, Dhana Thomy, Kirsten Famulla, Helena Castro, Peter Sass, Michael Groll, Heike Brötz‐Oesterhelt

**Affiliations:** ^1^ Interfaculty Institute of Microbiology and Infection Medicine Dept. of Microbial Bioactive Compounds University of Tübingen Auf der Morgenstelle 28 72076 Tuebingen Germany; ^2^ Laboratory of Antibiotics Biochemistry Education and Molecular modeling Department of Molecular and Cell Biology Federal Fluminense University Outeiro São João Batista, Centro Niterói 24210130 Rio de Janeiro Brazil; ^3^ Center for Integrated Protein Science at the Department of Chemistry Technical University Munich Lichtenbergstrasse 4 85748 Garching Germany; ^4^ Institute for Pharmaceutical Biology and Biotechnology University of Düsseldorf Universitätsstrasse 1, Building 26.23. 40225 Düsseldorf Germany

**Keywords:** acyldepsipepide, ADEP, antibiotics, ClpP, natural products, protease

## Abstract

Acyldepsipeptide (ADEP) is an exploratory antibiotic with a novel mechanism of action. ClpP, the proteolytic core of the caseinolytic protease, is deregulated towards unrestrained proteolysis. Here, we report on the mechanism of ADEP resistance in Firmicutes. This bacterial phylum contains important pathogens that are relevant for potential ADEP therapy. For *Staphylococcus aureus*, *Bacillus subtilis*, enterococci and streptococci, spontaneous ADEP‐resistant mutants were selected *in vitro* at a rate of 10^−6^. All isolates carried mutations in *clpP*. All mutated *S. aureus* ClpP proteins characterised in this study were functionally impaired; this increased our understanding of the mode of operation of ClpP. For molecular insights, crystal structures of *S. aureus* ClpP bound to ADEP4 were determined. Well‐resolved N‐terminal domains in the apo structure allow the pore‐gating mechanism to be followed. The compilation of mutations presented here indicates residues relevant for ClpP function and suggests that ADEP resistance will occur at a lower rate during the infection process.

## Graphical abstract

**ClpP mutant analysis**: ADEP antibiotics select ClpP out‐of‐function mutations in Firmicutes *in vitro*. Studying these ClpP defects provides a map of amino acids relevant for the function of ClpP. Several mutated ClpP proteins were purified, and their diverse impairments were characterised. A new *S. aureus* ClpP:ADEP co‐crystal structure shows an electrostatic network stabilising the N‐terminal gate.

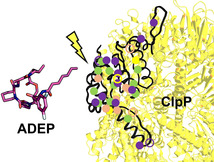

## Introduction

Antibiotic resistance spreads at an alarming rate.^1^ More than ever, it is essential to discover and explore new antibacterial scaffolds and mechanisms. Acyldepsipeptides (ADEP) represent a new class of antibiotics in preclinical development for the treatment of infections with multidrug‐resistant or persistent Gram‐positive bacteria. The class originates from a natural product complex produced by *Streptomyces hawaiiensis* NRRL 15010^2^, ^3^, ^4^ and synthetic derivatives with improved potency and metabolic stability were generated.^5^, ^6^, ^7^, ^8^, ^9^ ADEP demonstrated high efficacy *in vitro* and in curing rodent infections inflicted by *S. aureus*, enterococci and streptococci, including multidrug‐resistant strains and non‐growing cells.^10^, ^11^, ^12^, ^13^ ADEP acts by an unusual mechanism that does not require bacterial growth to take effect, hence its anti‐persister activity.^11^, ^13^ The antibiotic deregulates ClpP, the proteolytic core of the bacterial caseinolytic protease and a new target for antibiotic and anti‐virulence therapy.^10^, ^14^, ^15^, ^16^ ClpP is ubiquitous in bacteria, mitochondria, and chloroplasts.^17^, ^18^ In its active tetradecameric conformation, ClpP forms the central proteolytic compartment of a larger AAA+ protease machine.^19^ Two rings of seven ClpP monomers stack and interact to form a secluded barrel‐shaped tetradecamer harbouring the 14 catalytic sites.^20^, ^21^ Small entrance pores at the top and bottom of the barrel restrict access of proteins and allow isolated ClpP to degrade small peptides only.^22^, ^23^ For protein degradation, Clp‐ATPases are required (in Firmicutes ClpX, ClpC, and ClpE) to unfold designated substrates and thread them through the entrance pores.^16^, ^24^

ADEP was shown to bind to hydrophobic pockets at the surface of each heptamer ring, cavities that emerge when two adjacent ClpP subunits join.^25^, ^26^ By establishing contacts with both neighbouring ClpP monomers, ADEP stabilises the ring‐intrinsic interactions. The same hydrophobic pockets are essential contact points for the Clp‐ATPases.^27^, ^28^ ADEP was also shown to promote the interactions between the two distinct heptamer rings by driving ClpP into an extended conformation that is characterised by a stretched handle region (α5‐helix).^29^ Only in the extended tetradecamer conformation, the catalytic triad (Ser, His, Asp) of the serine protease has the right distance and orientation for catalysis.^20^, ^30^, ^31^ This way, ADEP stimulates the inter‐ring interactions and catalytic rate of ClpP. ADEP exerts further conformational control towards the hydrophobic N‐termini that line the pore of ClpP. The pore diameter is about 10 Å in apo‐ClpP and substantially widened in the ADEP‐bound state up to 30 Å.^13^, ^25^, ^26^, ^32^, ^33^ As a consequence, ADEP‐activated ClpP is capable of degrading polypeptides or flexible protein regions without the assistance of Clp‐ATPases.^10^, ^25^, ^26^, ^29^, ^34^, ^35^, ^36^

In terms of cell physiology, ADEP is bactericidal.^13^, ^37^ Bacteria react with a heat‐shock response indicative of the protein stress induced by degraded or truncated polypeptides and proteins.^10^ ADEP‐deregulated ClpP is capable of digesting nascent polypeptides emerging from the ribosome^34^ and the cell division mediator FtsZ emerged as a particularly sensitive protein target.^37^, ^38^ Deregulated proteolysis is the destructive power that kills Firmicutes during ADEP treatment *in vitro*,^35^ as under favourable *in vitro* conditions (nutrient‐rich, moderate temperatures, exponential phase), ClpP function is not essential in any of the Firmicutes species investigated so far.^15^, ^39^ However, it is crucial to keep in mind that during ADEP exposure – and based on the abrogated communication of ClpP with its cognate Clp‐ATPases – the natural functions of the Clp protease in Firmicutes are also prevented. Notably, lack of ClpP function, although not leading to a severe phenotype *in vitro*, was shown to have a substantial impact under infection conditions in the host.^14^, ^15^, ^40^, ^41^ In mycobacteria, where ClpP is essential under all conditions, it is the inhibition of the natural functions which causes bacterial death in the presence of ADEP.^35^

It is unusual for a bactericidal antibiotic to act on a non‐essential target, and this is only possible because the target protein can be diverted from its intended use and turned into a toxic instrument. A definite caveat is the issue that selection pressure applied to a non‐essential target causes rapid resistance development. Indeed, we and others have reported resistance rates in the range of 10^−6^ for *B. subtilis*, *Streptococcus pneumoniae* and *Enterococcus faecalis*, *in vitro*.^10^, ^11^, ^13^, ^42^ In the few cases where mutants were sequenced, spontaneous mutations had appeared in ClpP.^10^, ^13^, ^42^ Frameshift mutations and premature stop codons were reported, which result in nonfunctional nonsense proteins or truncations.^13^, ^42^ Promotor mutations observed can be easily explained by the lack of protein expression.^13^ It was also mentioned that substitutions of a single amino acid occur, but only one example was published (G127V in *B. subtilis* ClpP) and the characteristics of this protein were not investigated.^13^, ^42^

Here, we report on the functional analysis of several single amino acid mutations in different regions of *S. aureus* ClpP generated *in vitro* under selection pressure with ADEP. We focussed on *S. aureus* as a major target pathogen for potential future clinical application of the ADEP class but the findings presented here also shed light on ClpP mutations that we obtained in other genera of Firmicutes. To increase our understanding of the interaction between ADEP and its target in *S. aureus*, we also determined the crystal structure of ADEP4, a particularly potent ADEP congener, bound to *S. aureus* ClpP. In the high‐resolution structures presented here, the N‐terminal region of ClpP, which was often elusive in previous crystallographic efforts, is exceptionally well resolved. Further insights into the conformational states of the N‐terminal domain are provided and the hydrogen network that regulates those.

## Results

### ADEP frequently induces spontaneous *clpP* mutations *in vitro*.

**Figure 1:**
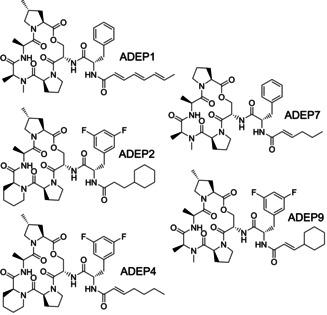
Structures of ADEP congeners used in this study. ADEP1 is a natural product of *S. hawaiiensis* and the most active among the main components of the natural product complex.^2^, ^3^ ADEP 2 to 9 are synthetic congeners.

In this study, we employed five different ADEP congeners, including the natural product ADEP1 from which the compound series originates and a range of synthetic derivatives presenting structural modifications in various regions of the molecules (Figure 1).

The ADEP series selected spans a broad potency range against *S. aureus*, from ADEP7 with a minimal inhibitory concentration (MIC) of 8 μg mL^−1^ to the optimised synthetic congeners ADEP4 and ADEP9 with MICs of ≤0.03 μg mL^−1^ (Table 1). Resistance rates, defined by the portion of bacterial cells capable of growing on ADEP‐containing agar, were in the range of 10^−6^ for all congeners tested and for two different *S. aureus* clinical isolates (Table 2).

**Table 1:** Minimal inhibitory concentration range for the ADEP series used in this study.

	MIC [μg mL^−1^]^[a]^					
	ADEP1^[c]^	ADEP2	ADEP4	ADEP7	ADEP9	Doxycycline^[d]^
*S. aureus* ATCC 29213^[b]^	1	0.125	0.031	8	≤0.031	0.06
*S. aureus* 133	0.5	0.031	≤0.031	4	≤0.031	0.06
*B. subtilis* 168	0.063	≤0.031	≤0.031	0.5	≤0.031	0.125
*E. faecalis* ATCC 29212^[b]^	0.063	≤0.031	≤0.031	2	≤0.031	4–8

[a] MICs determined by broth microdilution in Mueller‐Hinton broth for wild‐type strains. [b] Reference strains recommended by the Clinical Laboratory Standards Institute (CLSI) for quality control. See Table S1 for further information on strains used in this study. [c] Values for freshly dissolved ADEP1. [d] Used as a reference.

**Table 2:** Resistance rates^[a]^ of *S. aureus* clinical isolates.

	ADEP2		ADEP4		ADEP7		ADEP9	
ADEP exposure^[b]^	4× MIC	10× MIC	4× MIC	10× MIC	4× MIC	10× MIC	4× MIC	10× MIC
*S. aureus* ATCC 29213	7×10^−7^	7×10^−7^	9×10^−7^	9×10^−7^	6×10^−7^	n.d.	5×10^−7^	6×10^−7^
*S. aureus* 133	3×10^−6^	n.d.	2×10^−6^	n.d.	4×10^−6^	n.d.	4×10^−6^	n.d.

[a] Resistance rates were determined by plating bacteria on agar containing ADEP‐concentrations that prevented the growth of wild‐type cells (i.e., the fourfold or tenfold excess of the minimal growth inhibitory concentration). The number of resistant colonies was determined. For comparison, a resistance rate of 10^−6^ (i.e., one out of a million plated cells grows to a colony) is deemed high, 10^−8^ moderate and <10^−9^ rather low. [b] ADEP level applied in Mueller‐Hinton agar for selection of spontaneous resistance mutants.

**Figure 2:**
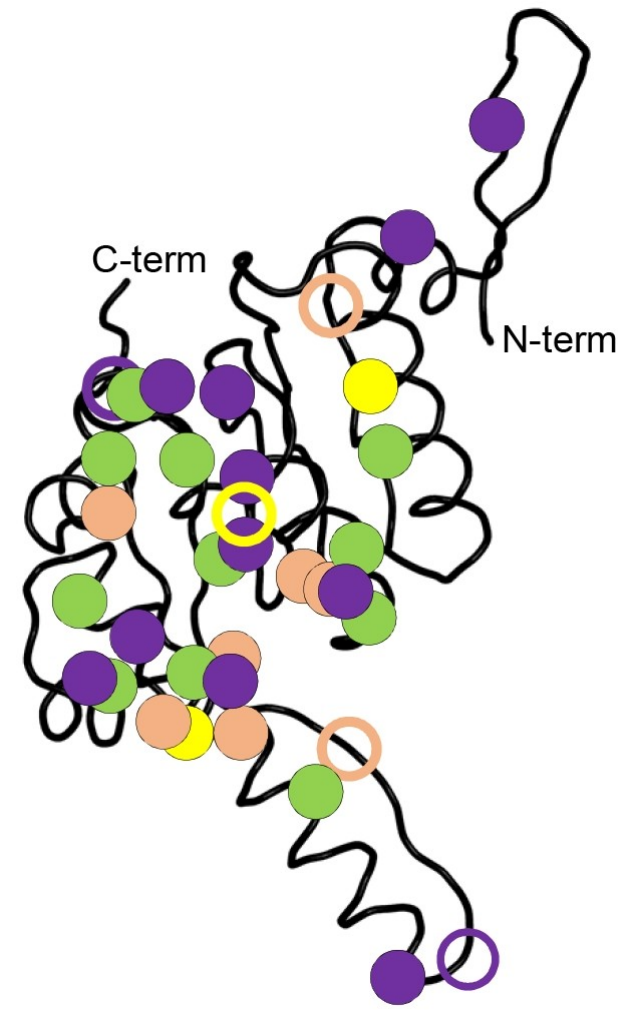
ClpP monomer with marked locations of amino acid exchanges conveying ADEP resistance. Colour code corresponds to the background colours in table 3. Filled circles indicate high‐level resistance. Hollow circles indicate intermediate resistance.

This result indicates that resistance development cannot be prevented by structural optimisation towards improved potency. Also, for *B. subtilis, Enterococcus faecium, E. faecalis, S. pneumoniae*, and *Streptococcus pyogenes* (then using ADEP1) we determined spontaneous resistance frequencies in the same range. A random selection of spontaneously resistant clones (RC) was picked from agar plates containing different strains and ADEP derivatives. Their MIC values were determined, and their *clpP* loci were sequenced, including the promotor region. Table 3 presents the broadest collection of *in vitro* selected ADEP‐resistant mutant clones across species to date. Without exception, all clones picked carried mutations in *clpP* and almost all of them were high‐level ADEP‐resistant. Mutations occurred all over the *clpP* gene (Figure 2). Insertions and deletions of single nucleotides appeared, leading to frameshifts and premature stop codons (Table 3). The latter were also generated by single nucleotide exchange, and we also observed a promoter mutation and a gene truncation. For these kinds of mutations, it seems evident that ClpP is out of function. Again, with a focus on *S. aureus*, we also tested genetically engineered ClpP deletion mutants that had previously been constructed in three different lineages (for strain details see Table S1). In the hospital‐acquired methicillin‐resistant *S. aureus* (MRSA) strain Col and the methicillin‐susceptible (MSSA) laboratory lineage 8325‐4, the *clpP* gene had been chromosomally deleted^43^, ^44^ and in the community‐acquired MRSA strain USA 300 JE2 it carries a transposon insertion.^45^ While all three wild‐type strains were highly susceptible (ADEP4 MICs≤0.03 to 0.06), all three corresponding Δ*clpP* strains were high‐level resistant (MIC>64 μg mL^−1^). This overview shows that (1) ClpP seems to be the only phenotypically relevant target of ADEP across all species tested and that (2) ClpP mutations occur as the, by far, most prominent cause of resistance, and probably the only cause of high‐level resistance, *in vitro*, in *S. aureus* as well as further Firmicutes.

**Table 3:** ClpP mutations detected in resistant clones (RC) generated by ADEP selection pressure.

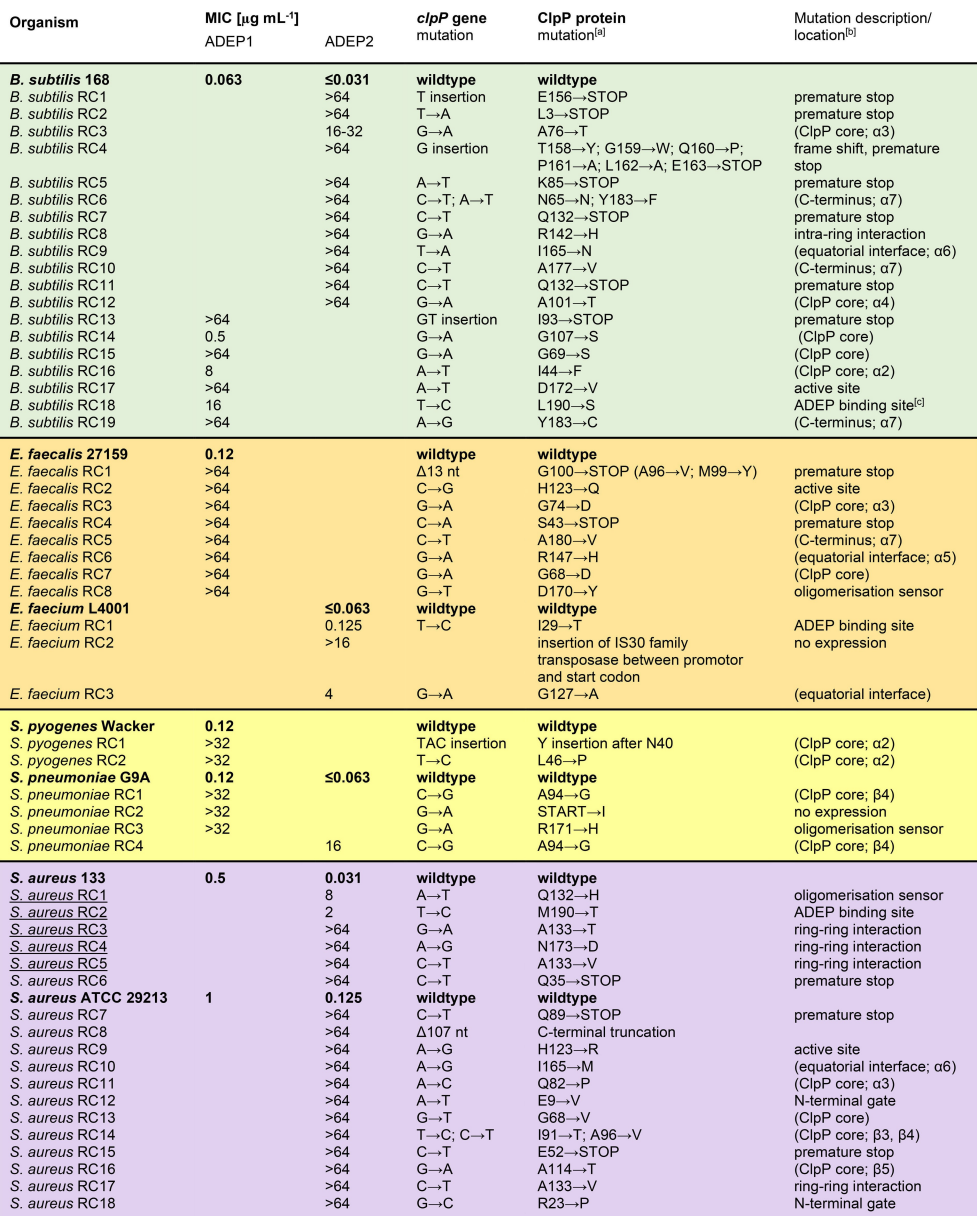

[a] Listed numbers are mapped to start methionine in position 1 for all species. [b] In cases, where the nature of the defect is obvious (e.g., premature stop) or where the mutation occurred in a region of known, or here confirmed, specific function (e.g., active site, ADEP binding site, oligomerisation sensor, N‐terminal gate, ring‐ring interaction, intra‐ring interaction), this information is provided here. Locations of mutations in less well characterised areas are depicted in brackets (e.g., ClpP core – i.e., globular head domain, equatorial interface and C terminus). Resistant clones (RC) are listed below the wild type from which they are derived. Mutants characterised in detail in this study are underlined. [c] For a depiction of the ADEP binding site see Figure S1.

### The pore opening mechanism of ADEP involves a functional network of electrostatic bonds at the N terminus of ClpP

**Figure 3:**
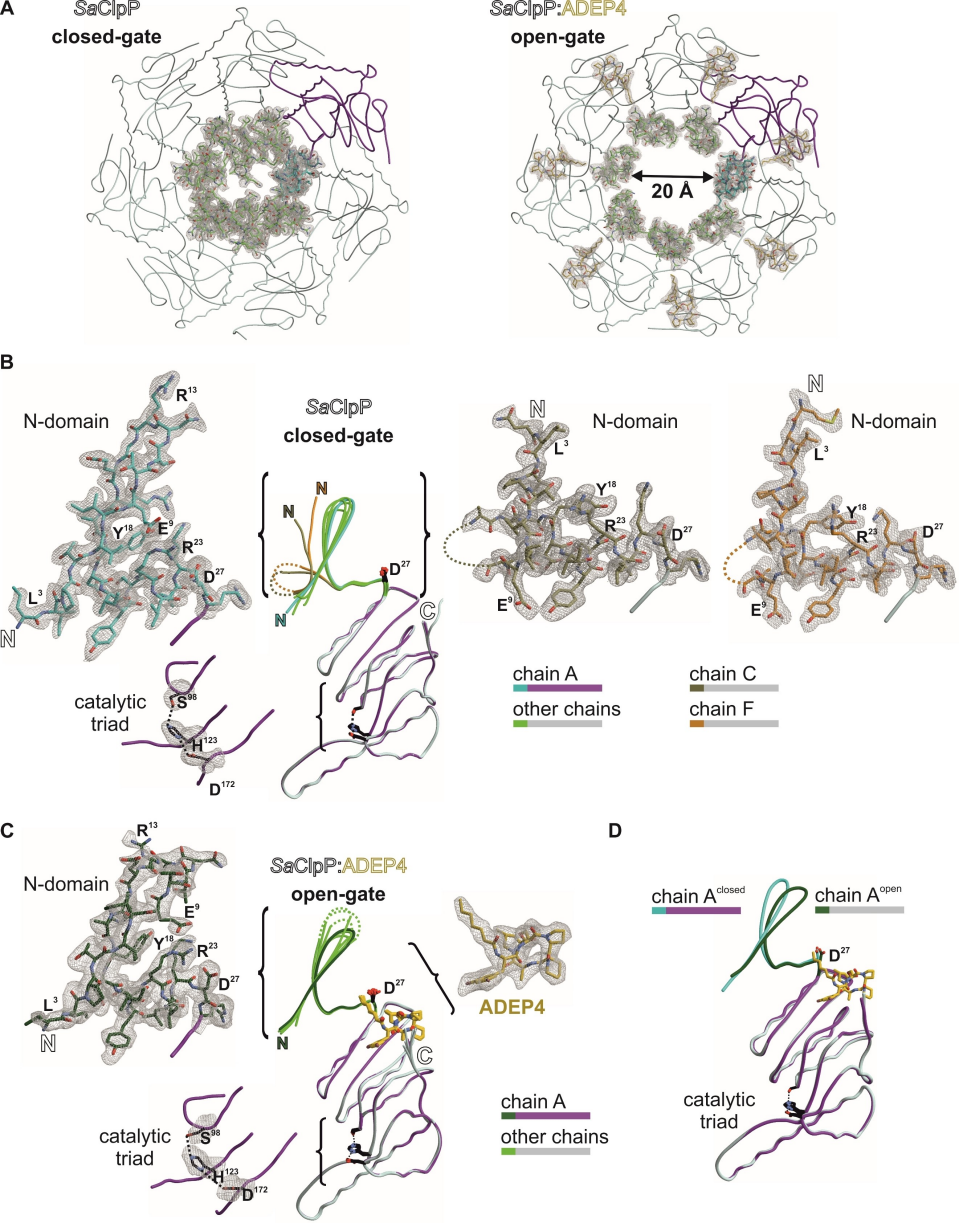
Crystal structures of SaClpP highlighting the electron‐density maps of the N‐terminal cluster. A) 2*F*_o_‐*F*_c_ simulated annealing composite omit electron‐density maps (contour level 1*σ*) of the α_7_‐ring in apo (closed conformation, PDB ID: 6TTY) and complexed with the acyldepsipeptide antibiotic ADEP4 (open conformation, PDB ID: 6TTZ). One subunit is coloured in magenta with its N‐domain (amino acids 1–28) in cyan. Other subunits are depicted in grey (N‐domains in green). B) and C) The N‐domain conformations in chains C (khaki) and F (orange) of the apo structure differ significantly from the other subunits and display a structural inversion; in contrast, the N‐domains in the ADEP4‐bound structure uniformly adopt the β‐hairpin. Disordered regions are displayed as dotted lines D) Structural superposition of chain A in closed‐gate and open‐gate structures.

At the time when this study was initiated, the only available ClpP:ADEP co‐crystal structure among Firmicutes was that of our non‐pathogenic model species *B. subtilis*. For *S. aureus*, a particularly relevant pathogenic target species of ADEP, only apo‐ClpP structures had been solved.^21^, ^31^, ^46^ In order to gain further insight into the interaction of ADEP with ClpP from *S. aureus* and thereby support our functional analyses of the mutated *S. aureus* ClpP proteins, we generated and solved the corresponding co‐crystal structure in conjunction with the apo‐protein at resolutions of 2.2 Å and 1.9 Å, respectively (Figure 3A). While typical features of ADEP‐bound ClpP structures from other organisms can also be found here,^8^, ^9^, ^13^, ^25^, ^26^, ^32^, ^33^ a specific feature of this SaClpP:ADEP4 structure are the well resolved N‐terminal β‐hairpin loops lining the axial pores (Figure 3C). This region of ClpP had remained unresolved in many previous structures due to the inherent flexibility of this area. Here, in contrast, residues 4 to 10 and 16 to 20 are well defined in all seven subunits. For the first and sixth subunit within the heptamer ring (chain A and F in PDB 6TTZ) even the entire loop could be resolved with exceptional electron density (Figure 3B), clearly displaying the β‐hairpin element that forms the outermost N‐terminus of ClpP.

**Figure 4:**
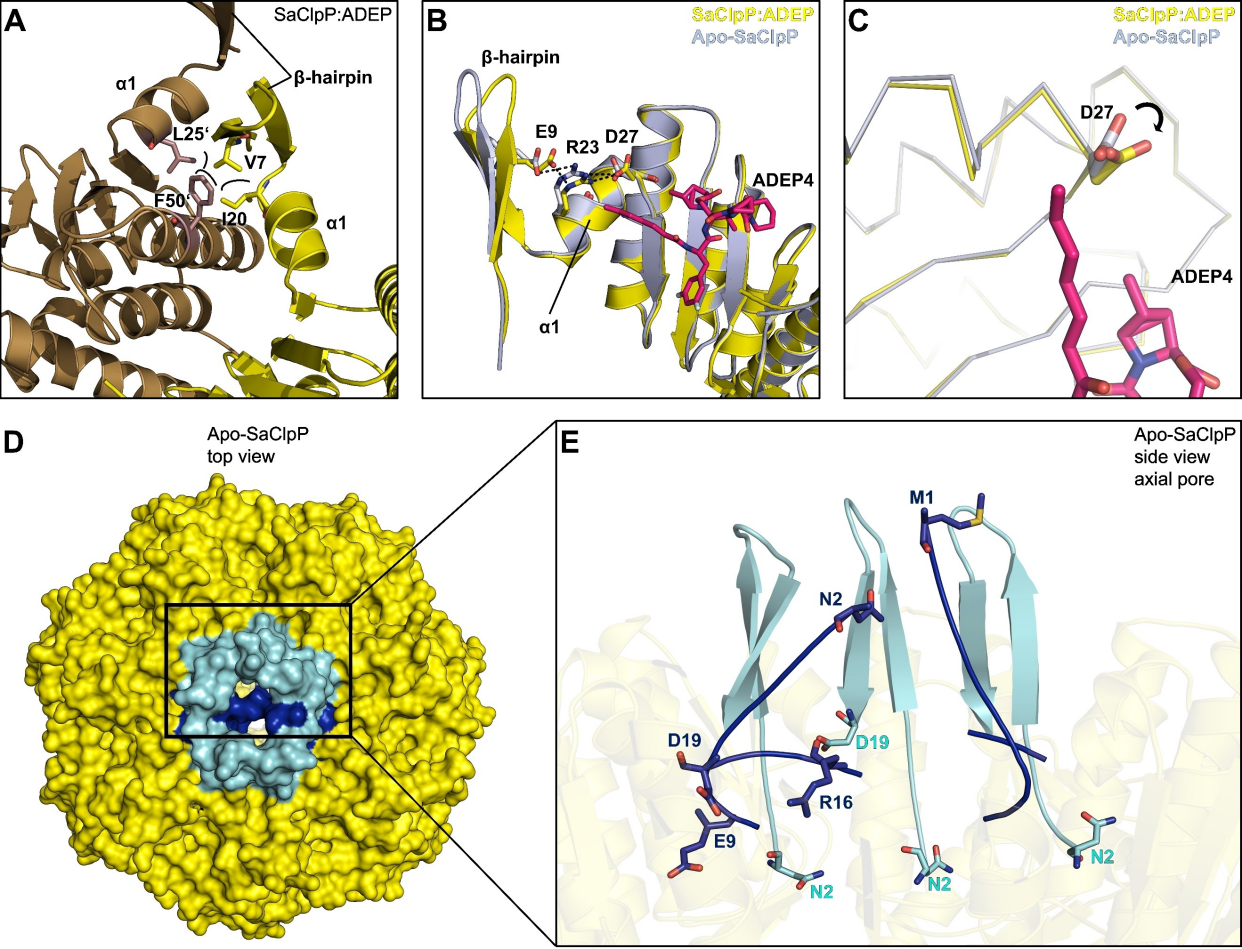
Crystal structures of SaClpP in its apo (PDB ID: 6TTY) and ADEP4‐bound (PDB ID: 6TTZ) forms with focus on the N‐terminal cluster. A) Inter‐subunit connection: depiction of N‐terminal hydrophobic interactions mediating inter‐subunit stability and β‐hairpin orientation. Two adjacent ClpP subunits are depicted in yellow and brown. B) Intra‐subunit connection: Overlay of the N‐terminus of SaClpP:ADEP4 (yellow) with the N terminus apo‐SaClpP in a β‐hairpin orientation (grey). Key residues of the N‐terminal hydrogen‐bonding network display a shift in orientation that propagates to the axial pore. C) Rotation of the D27 residue upon binding of ADEP4. D) Top view of the apo‐SaClpP crystal in a surface representation. N‐terminal β‐hairpin regions (residues 1–19) that adopt the “up conformation” are coloured cyan, corresponding regions that adopt the “down conformation” are coloured dark blue. E) N‐terminal β‐hairpin regions of five ClpP subunits highlighted in a close‐up view of the axial pore. Three out of five of these regions are in the up conformation (cyan) and two in the down conformation (dark blue). The denomination “up” refers to the β‐hairpin loop. N‐terminal regions that adopt the up conformation form a well‐defined, upwards pointing β‐hairpin structure and have their N‐termini (see N2) facing downwards. The two subunits adopting the down conformation are disordered and have their N termini facing upwards (see M1, N2) with the residues E9 to D19 extending into the axial channel and contributing to pore closure.

Two interactions seem critical in anchoring the N‐terminal β‐hairpin loops in position. V7 forms close hydrophobic contacts with F50’ and L25’ from the adjacent subunit as well as I20 from the same subunit and stabilises the N‐terminal domain by inter‐subunit interactions (Figure 4A). The importance of this particular residue for the barrel integrity of SaClpP has been previously demonstrated by the finding, that the V7A mutant displays a remarkable split‐ring conformation where one subunit of a given ClpP ring adopts a compressed rather than extended conformation resulting in a coiled structure spiralling around the rotary axis.^47^ Furthermore, the corresponding V6 residue of *E. coli* ClpP has been shown to be essential for the interaction with Clp‐ATPases but was structurally similar to wild‐type and catalytically unimpaired.^48^ The second important interaction is formed by a network of electrostatic bonds previously described to be involved in the formation and orientation of the N‐terminal β‐hairpin structure.^26^, ^33^ In this context, the amino acid R23 is of particular importance (Figure 4B) and plays a special connecting role by establishing one hydrogen bond to E9 of the N‐terminal cluster (N‐domain; M1‐D19) and another to D27 of α1‐helix of the ClpP main body. D27 is in the direct vicinity of the outermost end of the aliphatic tail of ADEP4, corroborating a previous observation of Mabanglo and co‐workers where the corresponding *E. coli* ClpP residue E40 formed a hydrophobic interaction with the tail of bound ADEP1.^33^ Binding of ADEP initiates rotations at the key residues of the hydrogen network. An overlay of the D27 moieties of all ClpP subunits within one apo‐ClpP ring shows a stable conformation in suitable H‐bond distance to R23, while upon insertion of the hydrophobic ADEP tail region this order is disturbed (Figure S3). In addition, D27 turns towards the main body of ClpP and R23 as well as E9 follow suit, thereby reorienting the N‐terminal β‐sheet that is now slightly tilted by 15° (Figure 4B). The rigidity of the nonpolar side chain of ADEP4 induces a reorientation of the hydrophilic D27 carboxylate, which is the terminal amino acid of helix α1. As illustrated in the enlarged section (Figure 4B, C), this tilt causes a marginal twist of helix α1, thereby shifting the guanidino group of R23 by two angstroms. This generated molecular switch ultimately leads to rearrangements of the unconstrained axial loop stabilising the open gate. Altogether, the structural shifts between the two well‐defined β‐hairpin structures of apo and liganded SaClpP are marginal, and the pore opening upon ADEP binding is primarily achieved by an outward movement of the entire N‐domain (see Figure 3A, also discussed later in this chapter). Interestingly, two spontaneous mutations that were selected in SaClpP by ADEP exposure (Table 3) relate to these residues. Exchange by mutation of E9 and R23 to valine and proline, respectively, mediate high‐level ADEP resistance (see RC12 and RC18 in Table 3), probably through the loss of the pore controlling functionality that depends on the well‐defined electrostatic interactions formed by these residues. In the aforementioned publication by Mabanglo et al., crystal structures of ClpP from *E. coli* and *Neisseria meningitidis* bound to ADEP and ACP activators were solved. When the authors mutated E40 (EcClpP) and E31 (NmClpP), both of which correspond to D27 of SaClpP, to alanine, they observed a moderate gain‐of‐function phenotype that allowed a full‐length protein substrate (i.e., casein) to be degraded to some extent in the absence of activator compounds.^33^ However, when we mutated E9, R23, and D27 to alanine in SaClpP, oligomeric integrity of the SaClpP tetradecamer was lost, resulting in a loss of catalytic function (Figure S4). Still, the addition of ADEP4 could reactivate either of these alanine mutants and enabled them to degrade casein which requires active pore opening (Figure S4). This result implies that although important for the ADEP effect as demonstrated by the emergence of the two highly resistant clones E9V and R23P, exchanging these key residues to alanine might still leave ADEP with sufficient capacity of opening the pore. Interestingly, among the four N‐terminal gating residues presented in Figure S4, only the E9A mutant retained residual activity in a SaClpXP‐dependent GFP‐ssrA degradation assay. Recently published ClpXP cryo‐EM structures suggest that β‐hairpins form in the N‐terminal domain of ClpP upon interaction with ClpX.^49^, ^50^ In conjunction with previous findings stating that the corresponding E8 residue in *E. coli* ClpP only modestly contributes to the formation of the electrostatic network of ClpP, it can be assumed that while important for the interaction with Clp‐ATPases, the formation of a structured β‐hairpin relies more strongly on the respective contributions of the R23 and D27 residues than on the E9 moiety.^51^

The well‐resolved N‐terminal domains within the apo‐SaClpP crystal structure (PDB ID:6TTY) display a conformation not described for SaClpP, so far. Two distinct conformations could be clearly differentiated within the N terminus of ClpP (Figures 3B and 4D,E). Five out of seven subunits display a well‐defined upwards pointing β‐hairpin loop and downwards facing N termini. In contrast, in the remaining two, a structural inversion of the first nineteen amino acids can be observed (Figures 3B and 4E). This inversion leads to the loss of the structured hairpin and the respective N termini are facing upwards with a slight tilt towards the barrel rotation axis. The residues E9 to D19 form a loop that extends into the axial channel and, in conjunction with the terminal residues M1 to V7, facilitate pore closure. In line with the previously solved ClpP structures displaying an intra‐subunit strengthening of the electrostatic interaction network upon activator binding, our structures confirm that binding of ADEP not only causes the uniform adoption of a β‐hairpin (Figure 3C) in all the ClpP subunits but also an outward movement of the entire N‐terminal domain (Figure 3A).^8^, ^9^, ^13^, ^25^, ^26^, ^32^, ^33^, ^52^, ^53^ These two structural shifts are jointly responsible for the effective increase in pore diameter (Figure 3A).

### ADEP‐induced ClpP mutations impair the function of the Clp protease machine

Apart from ADEP resistance mutations where ClpP protein truncations made loss of function apparent, there were also a substantial number of single amino acid exchanges, where ClpP functionalities could not be reliably predicted. Towards a better understanding of such mutated ClpP proteins selected under ADEP pressure, we overexpressed and purified five full‐length *S. aureus* ClpP variants carrying single amino acid exchanges in different regions of the protein. These five proteins were selected for the following reasons: Among the 18 *S. aureus* mutant clones picked, only two (Q132H, M190T) had some remaining sensitivity to ADEP on the MIC level. Their ClpP proteins were included, as we hoped to find at least residual proteolytic capabilities. The position A133 was mutated in three of the 18 clones, being the most often mutated moiety in the SaClpP panel and having emerged in two strain backgrounds and with two different amino acid exchanges (A133T, A133V). Both SaClpP A133 variants were selected to compare their performance. The fifth mutant protein chosen for in‐depth analysis was N173D, because this mutation had emerged in the same strain background (*S. aureus* 133) as the four ones above.

**Figure 5:**
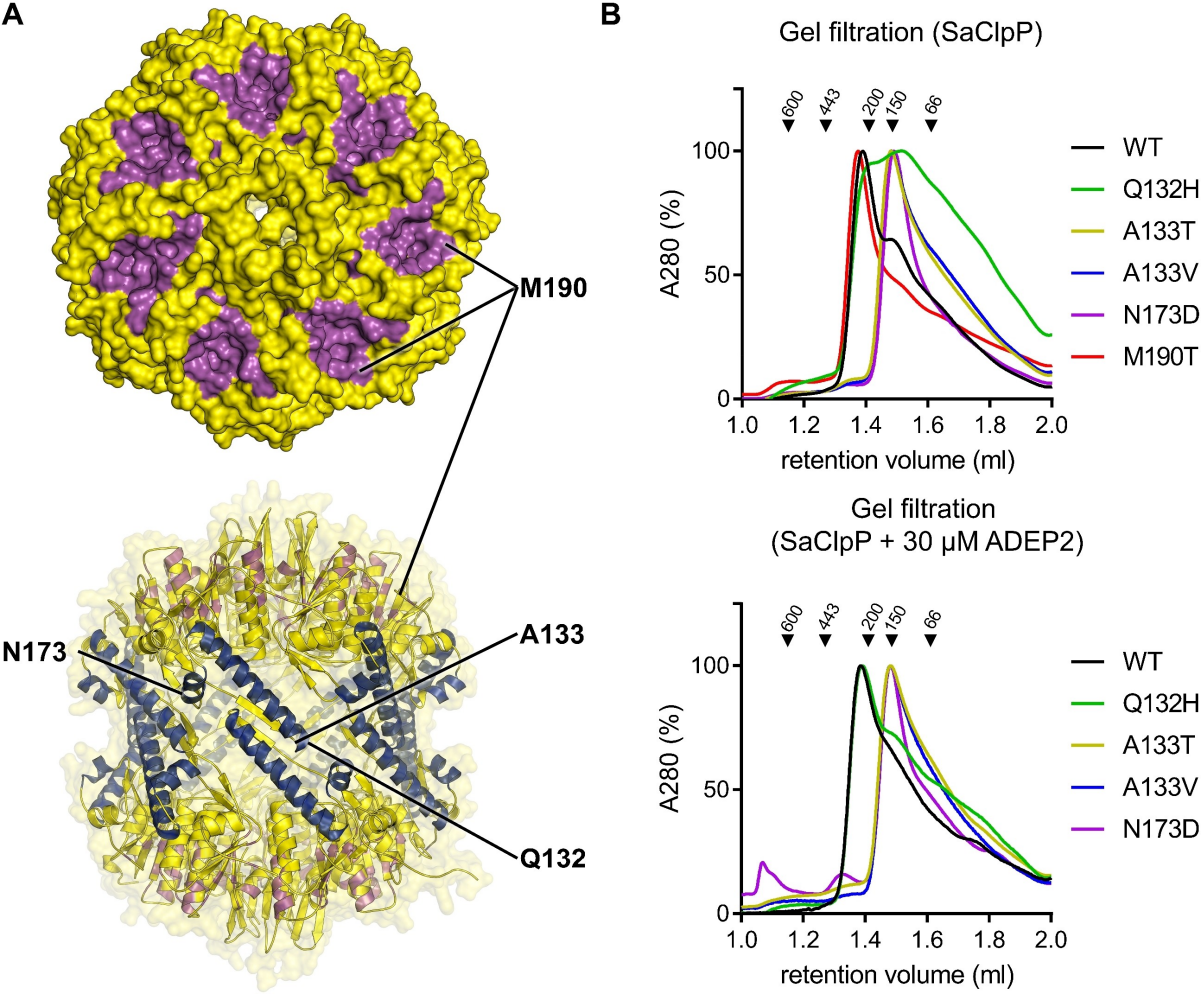
Analysis of the oligomeric state of mutated *S. aureus* 133 ClpP proteins selected under ADEP pressure. A) Positions of amino acid exchanges within ClpP pictured with the help of the apo‐SaClpP crystal structure (PDB ID: 6TTY) solved in this study. The top panel shows a top‐view surface representation of SaClpP with the ADEP binding sites coloured in magenta. The lower panel depicts a side‐view cartoon representation with the α5‐ and α6‐helices highlighted in dark blue. B) Gel filtration analysis of purified SaClpP mutant proteins. In the absence of ADEP, all but the M190T mutant show a defect in tetradecamer (301 kDa) formation. The Q132H mutant forms a mixture of different oligomeric states ranging from full tetradecamer to monomer (21.5 kDa) but can be transformed into a tetradecamer by the addition of ADEP. The mutants A133T, A133V, and N173D adopt a heptameric (150.5 kDa) conformation both in the absence and presence of ADEP. At an even higher ADEP concentration (60 μM), the mutants A133T, A133V and N173D showed a small proportion of tetradecamer (Figure S5). To assess the effect of ADEP, the proteins were preincubated with ADEP before being subjected to size‐exclusion chromatography, but the chromatography buffer contained no ADEP.

**Figure 6:**
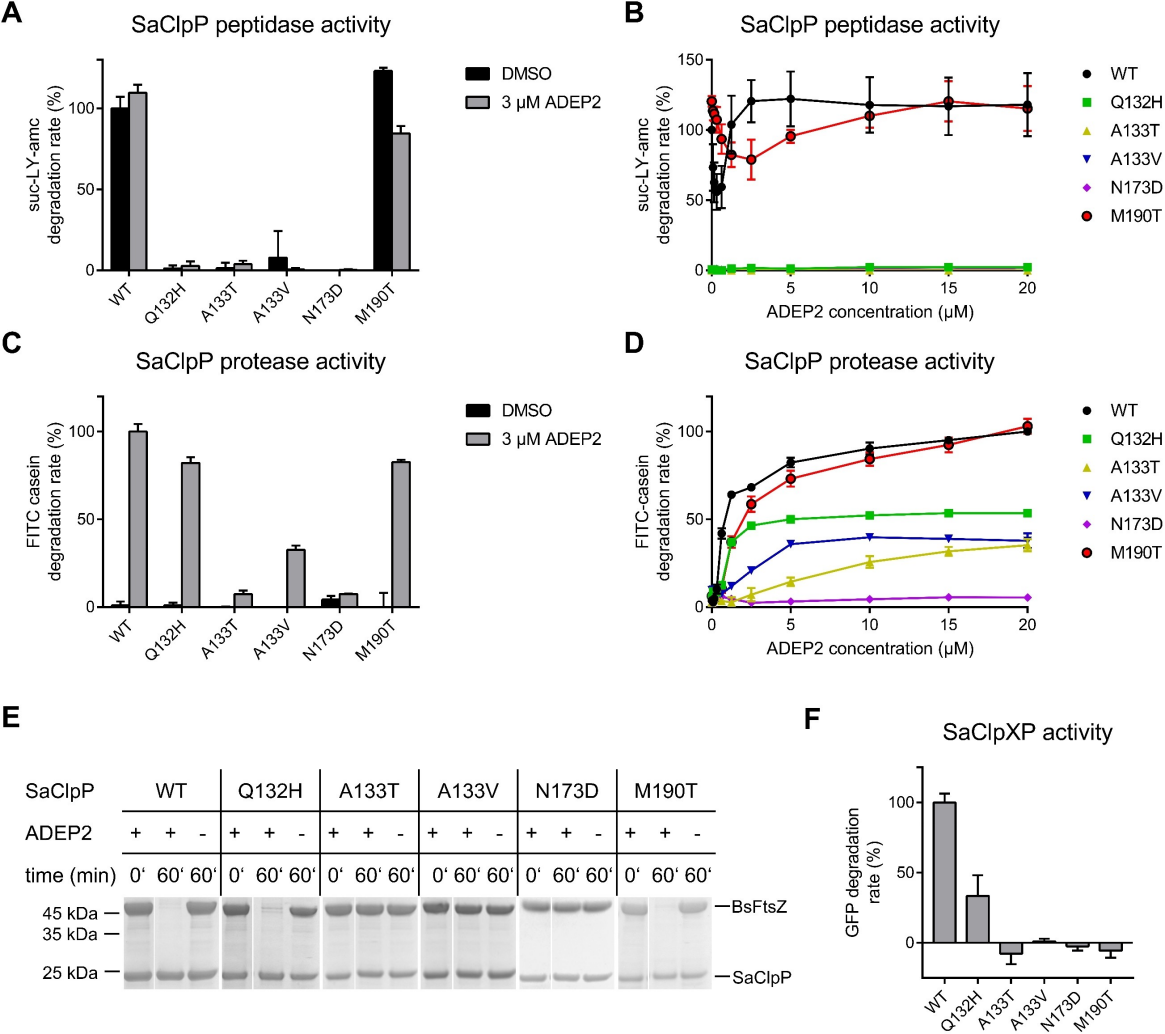
*In vitro* degradation assays with ADEP‐resistant *S. aureus* 133 mutants RC1‐5. A) Degradation assays of fluorogenic model peptide substrate Suc‐LY‐AMC by 1 μM of SaClpP in the presence and absence of 3 μM ADEP2. 3 μM ADEP2 is sufficient to fully stimulate SaClpP for peptide degradation (compare panel B). Error bars indicate S.D. (three independent experiments). B) Suc‐LY‐AMC degradation assays across an ADEP2 concentration range of 0–20 μM (1 μM of SaClpP). Error bars indicate S.D. (three independent experiments). C) Degradation assays of the fluorogenic model full‐length protein substrate FITC‐casein by 1 μM of SaClpP in the presence and absence of 3 μM ADEP2. Error bars indicate S.D. (three independent experiments). D) FITC‐casein degradation assays across an ADEP2 concentration range of 0–20 μM (1 μM of SaClpP). Error bars indicate S.D. (three independent experiments). E) Degradation assays of 5 μM of purified FtsZ protein (from *B. subtilis*) by 2.5 μM of SaClpP in the presence and absence of 6.25 μM ADEP2 analysed by SDS‐PAGE over 60 min. F) Degradations assays of 0.36 μM of purified eGFP tagged with a ssrA degradation tag. Degradation was signalled by the loss of GFP fluorescence. SaClpP and SaClpX were mixed at stoichiometric concentrations (0.2 μM of SaClpP_14_, 0.4 μM of SaClpX_6_). Error bars indicate S.D. (three independent experiments).

All five mutants were characterised for their oligomeric state, peptidolytic and proteolytic capacity as well as their responsiveness to the activators ADEP and ClpX. In Table S2, a comparative summary of the mutant profiles is provided. The frequently mutated A133 moiety is located at the very tip of the α5 helix of SaClpP (Figure 5A), also denoted as the handle region. Both mutated variants presented themselves as heptamers after purification (Figure 5B). Accordingly, they were catalytically inactive, as demonstrated by their failure to degrade the fluorogenic model peptide substrate Suc‐Leu‐Tyr‐AMC (Suc‐LY‐AMC; Figure 6A,B). Low residual casein degradation activity could be triggered by ADEP addition and further stimulated at high ADEP levels, but proteolysis values remained far below that of the wild type (Figures 6C,D and S5). The capacity of ADEP to stimulate the catalysis of particular ClpP mutant proteins is known and based on the fact that ADEP stabilises ClpP in the extended conformation. Shifting the equilibrium to the extended state promotes inter‐heptamer interactions and catalytic turnover.^16^, ^29^

Indeed, when pre‐treated with a high concentration (60 μM) of ADEP2 and then subjected to size‐exclusion chromatography, both A133 mutants showed a small tetradecamer fraction (Figure S6). To put this into perspective, for wild‐type SaClpP, 3 μM ADEP2 was sufficient for full catalytic stimulation (Figure 6B) and to achieve an almost maximal casein degradation rate (Figure 6D). It is also important to note that during ADEP‐mediated casein degradation, pore opening is rate‐limiting and not catalytic turnover (compare Figure 6C and 6A, both generated at the same ADEP2 concentration of 3 μM). Further profiling showed that both A133 mutants failed to degrade the cell division protein FtsZ in the presence of ADEP (Figure 6E), neither was ClpX capable of activating them for productive degradation of ssrA‐tagged GFP (Figure 6F). On the whole, both A133 mutant proteins are almost entirely inactive, with the nonpolar to polar A133T exchange generating an even more severe defect than the conservative A133V. The small proteolytic effects that we were able to produce in the presence of elevated ADEP concentrations *in vitro* seem of low relevance in the whole‐cell setting, as both A133 mutant clones showed ADEP MICs of >64 μg mL^−1^ (Table 3).

The next mutant investigated carried an amino acid exchange in the adjacent position (Q132H). The corresponding protein adopted a broad range of oligomeric states, from tetradecamer to monomer. Its tetradecameric fraction eluted from the size‐exclusion column slightly later than the wild‐type protein, in a non‐functional conformation (Figures 5B and 6A,B). Despite being catalytically impaired (1–2 % residual peptidase activity) and in contrast to the A133 mutants presented before, Q132H could still be substantially activated by ADEP for the degradation of casein (80 % of wt level, Figure 6C) as well as FtsZ (Figure 5E), in accordance with the still detectable antibacterial activity against the corresponding *S. aureus* clone RC1 (MIC 8 μg mL^−1^, Table 3). Also, in the Q132H mutant, ADEP induced the tetradecameric state and at 30 μM ADEP a fraction of mutated protein eluted with the same retention time as the wild type. The results observed for this mutant correspond well to a previously described mutant in the same position (Q132A), which had also eluted in lower oligomeric states and had lacked peptidase activity.^21^ The Q132 residue was previously proposed to play an important role in a hydrogen and electrostatic network (involving D170, R171, E135, and Q132 and denominated “oligomerisation sensor”) that mediates the interaction between the two heptameric rings as well as adjacent subunits within the same ring.^21^, ^31^ The oligomerisation defect of the current Q132H mutant corroborates this notion. Considering the central roles of Q132 and E135 in the oligomerisation sensor, it is not astonishing that mutating the A133 moiety also disturbs the inter‐ring interaction. Although A133 itself does not take part in the salt‐bridge network, molecular dynamics simulations distinguished this residue as the one with the largest r.m.s.f. value (i.e., root mean square fluctuation as a measure of flexibility) within the entire ClpP protein.^31^ Located at the very tip of the α5 helix, the A133 and Q132 represent the outermost moieties within the highly flexible sequence H123 to L145 that undergoes dynamic stretching and bending. In another mutant, the N173D mutation of SaClpP occurred within the α6 helix (Figure 5A). This position is located next to the active site aspartate D172 and in close vicinity to D170 and R171 of the oligomerisation sensor. N173 has not been characterised biochemically before. The profile of N173D is characterised by a heptameric state (Figure 5B) and a lack of activity under all conditions tested here, even including exposure to high ADEP concentrations (Figure 6B–F). It is conceivable that this deficiency is based on a disturbance of the oligomerisation sensor.

Among the *S. aureus* mutants isolated, M190T is the one still most responsive to ADEP (MIC *S. aureus* RC2: 2 μg mL^−1^). The mutation is positioned at the outer margin of the ADEP binding site (hydrophobic pocket, Figure 5A; Figure S1), thereby reducing ADEP affinity (Figure 6B) but not abolishing ADEP binding. The oligomeric state of the protein is an intact tetradecamer and only slightly higher ADEP concentrations are required for wild‐type‐like casein degradation rates (Figure 6D). FtsZ degradation works with ADEP‐activated M190T as efficiently as with wild‐type ClpP (Figure 6E). The most remarkable feature of this ClpP variant is that ClpX responsiveness is completely abolished in the concentration range tested (Figure 6F). This result concurs with our previous observation that the affinity of ADEP for the hydrophobic pocket of ClpP is higher than that of ClpX and that a single ADEP molecule is sufficient to displace an entire ClpX ring from ClpP.^29^ This inhibition of protein‐protein interaction was demonstrated for all Clp‐ATPases tested, so far, and a range of organisms.^25^, ^32^, ^34^, ^35^, ^36^ A general conclusion might be that most (if not all) mutations of the ADEP binding site impair the interaction of Clp‐ATPases with ClpP and, thus, the physiological functions of the Clp protease machine. Lack of Clp protease function in *S. aureus*, either by chemical inhibition of ClpP or by knock‐out, was previously shown to reduce the excretion of virulence factors, such as hemolysins, and to increase susceptibility to hydrogen peroxide.^43^, ^54^, ^55^ To investigate if our ADEP resistance clones RC1 to 5 were impaired in the same way, we performed a haemolysis and a hydrogen peroxide disc diffusion assay. While the *S. aureus* 133 parent strain was surrounded by a clear haemolysis zone on blood agar, all of its five ClpP mutant clones lacked haemolysis (Figure S7). In addition, RC1 to RC5 were also more susceptible to reactive oxygen species than the wild type, which might indicate reduced intra‐macrophage survival of the mutants (Figure S8).

### ADEP resistance can also be mediated by ClpP hetero‐tetradecamer formation

All ADEP‐resistant clones isolated in this study had mutations in *clpP*. We asked ourselves if there could potentially also be a secondary, less relevant target of ADEP that we had no chance to detect because *clpP* mutated so frequently. To explore this hypothesis, we introduced two additional IPTG‐inducible ectopic copies of the *clpP* gene into the *lacA* and *aprE* loci of the *B. subtilis* 168 chromosome, assuming that the presence of the three identical *clpP* genes would take the selection pressure away from *clpP*. Rationalising further that ADEP is deadly as long as there is functional ClpP, we expected the resulting mutant *B. subtilis* 3x*clpP* to show a much lower resistance mutation frequency. We were astonished that the resistance rate for the engineered strain was still 10^−6^ on ADEP containing agar plates and that sequencing revealed *clpP* to be still mutated. Depending on the location of the mutated *clpP* gene, analysed *B. subtilis* 3x*clpP* clones showed different MIC values for ADEP2. When the mutation resided in the original locus, cells showed high‐level resistance (>64 μg mL^−1^), while mutations in the ectopic loci only displayed MIC values in the range of 1 μg mL^−1^.

**Figure 7:**
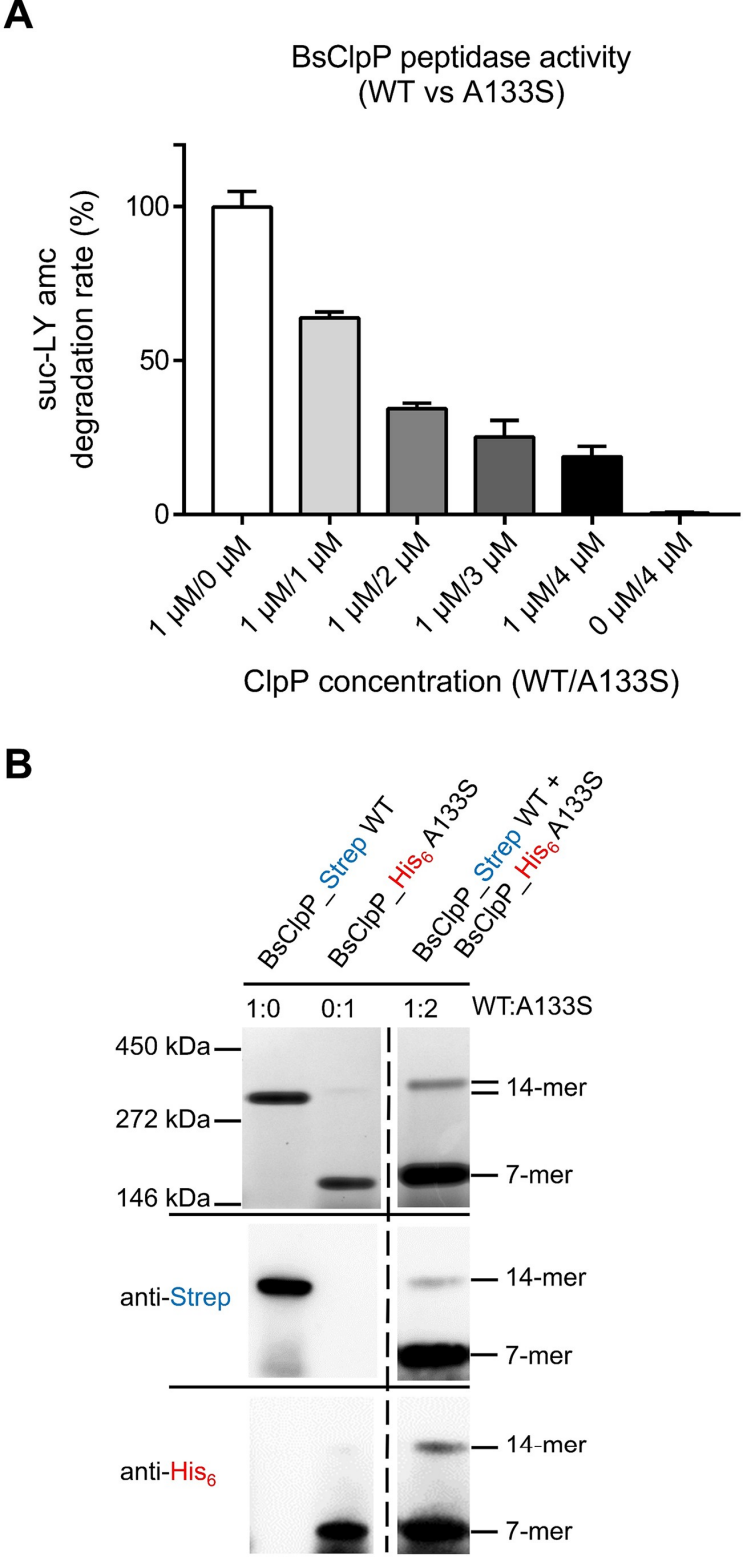
Purified ClpP proteins of *B. subtilis* wild type (BsClpP_WT) and its A133S mutant (BsClpP_A133S) form mixed, inactive complexes. A) Peptidase activity upon mixing both ClpP variants. The catalytic activity of ADEP‐activated BsClpP_WT (amount 1 μM kept constant in all samples) decreased when rising amounts of BsClpP_A133S (1 to 4 μM) were added. ADEP was present at 10 μM in all samples. Error bars indicate S.D. (three independent experiments). In a control experiment, bovine serum albumin was added to BsClpP_WT, without effect (Figure S9). B) Native PAGE and immunoblot analysis (employing anti‐Strep and anti‐His_6_ antibodies) of a mixture of BsClpP_WT (C‐terminal Strep‐tag) and BsClpP_A133S (C‐terminal His‐tag). When analysed individually, BsClpP_WT and BsClpP_A133S were used at 7 μM final concentration. When co‐incubated, 14 μM BsClpP_A133S was mixed with 7 μM BsClpP_WT. In all samples, ADEP2 was applied in a fourfold molar excess over total ClpP. Native PAGE and western blot images are representative of three biological replicates. For comparison, the oligomeric state in the absence of ADEP is shown in Figure S9.

Thinking this over, it occurred to us that although *B. subtilis* 3x*clpP* carried the same coding region of *clpP* in all three cases, the promoters were different. In the two ectopic loci, *clpP* was expressed from an IPTG‐inducible promoter, whereas it was behind its native promoter in the native locus. And indeed, comparison of the ClpP protein expression levels provided the first clue. ClpP was well expressed from the two ectopic loci, raising the ClpP level in *B. subtilis* 3x*clpP* in the presence of IPTG substantially above wild type (Figure S9). We could also see that ClpP produced from these sites was functional, as the susceptibility of IPTG‐induced *B. subtilis* 3x*clpP* to ADEP was eight times higher than that of the parental wild type, in accordance with the elevated concentration of the deadly target. However, when *B. subtilis* 3x*clpP* was exposed to ADEP, expression from the native locus by far exceeded the expression from both IPTG‐induced loci combined (Figure S9). Here, we observed the strong induction of the native *clpP* locus as part of the heat‐shock regulon when the cell encountered ADEP‐mediated protein stress. The fact that ADEP is capable of increasing the level of its target had also been observed before.^10^ At this point, we knew that mutated ClpP proteins originating from the native locus would be present in much higher quantities than such emerging from one of the ectopic loci. But we still did not know, why the two intact *clpP* gene copies that were still present in all ADEP‐resistant *B. subtilis* 3x*clpP* mutant clones could not kill the cell. In principle, they should have provided enough functional target to ADEP. Next, we purified native ClpP (BsClpP_WT) as well as a mutated ClpP protein (BsClpP_A133S) that had resulted in high‐level ADEP resistance when expressed from the native locus. Of note, the amino acid position A133 was also mutated in three of our *S. aureus* clones (Table 3). In biochemical assays, we then titrated the two *B. subtilis* ClpP proteins against each other and noticed that the peptidase activity of BsClpP_WT steadily decreased in a stoichiometric ratio with increasing concentrations of BsClpP_A133S (Figure 7A). We also analysed the oligomeric state of the proteins alone and in combination by native page and immunoblot using C‐terminally Strep‐ and His‐tagged proteins to allow the detection of both variants in the mixed situation. BsClpP_WT formed a high molecular weight band in the presence of ADEP2 indicating formation of a tetradecamer, while BsClpP_A133S formed a heptamer (Figure 7B), in accordance with the oligomerisation defect that we had detected in the corresponding *S. aureus* mutant (Figure 5B). When BsClpP_A133S was in twofold molar surplus over BsClpP_WT, almost all of the wild‐type protein was shifted to a mixed heptamer. Extended exposure of the blot also revealed a faint tetradecamer band that represented a mixed complex of both ClpPs. Hence, ADEP resistance in *B. subtilis* mutants, where only one of the three *clpP* gene copies was mutated, can be explained by the formation of an inactive heterocomplex of wild type and mutated ClpP.

## Discussion

This study was performed with two central aims. First, to use ADEP‐resistant mutants as a tool to improve our understanding of the operation mode of ClpP and the mechanistic details of its deregulation by ADEP. Second, to study the nature of the mutations selected by ADEP exposure and learn about the implications for future ADEP therapy.

Towards our first aim, we characterised the biochemical functionalities of five ADEP‐resistant *S. aureus* ClpP proteins and could highlight the importance of several amino acids for the operation mode of ClpP. For the Q132 residue, which had been reported to take part in the oligomerisation sensor,^21^, ^31^ we confirm the central role in establishing inter‐ring interactions. As noted before,^21^ we found the Q132 mutant to be strongly impaired in catalysis. However, the strong activator ADEP, and to a smaller extent also ClpX, were still capable of activating Q132H for substantial proteolysis (Figure 6). Position A133 was mentioned before as being exposed at the outermost tip of the α5‐helix and as being the most flexible residue within the entire ClpP protein,^31^ but it had not been characterised biochemically. We found this amino acid to be essential for inter‐ring interaction (Figure 5), and not even ADEP could elicit more than a small fraction of protein degradation from this mutant (Figure 6). It is noteworthy that this position was mutated three times in our mutant panel, in two different *S. aureus* lineages and in *B. subtilis* (Table 3, Figure 7). We also demonstrate an inter‐ring oligomerisation defect for the N173D mutant (Figure 5). Here, the affected position is next to the active site aspartate and the oligomerisation sensor.

ADEP can promote the conversion of inactive ClpP variants towards active tetradecamers, which was clearly visible for some mutants and corroborates the strong conformational control that the antibiotic exerts over ClpP. Previously, we had observed that ADEP converted the D172N mutant of SaClpP, which had been in the catalytically inactive state of a compressed tetradecamer, to the active extended tetradecamer conformation.^29^ In the current study, we noted ADEP's tetradecamer stabilising potential particularly for the Q132N mutant (Figures 5 and 6) and several mutants of the N‐terminal network (Figure S4). ADEP assembled heptamers and even lower oligomers to the respective tetradecamers. The antibiotic exerts conformational control over ClpP in two different directions. On the one hand, over long distance along the vertical axis of ClpP, with the consequence of shifting the dynamic equilibrium towards the extended conformation and stabilising the catalytically competent spatial arrangement of the active sites.^16^ In addition, at the apical surface, conformational changes are propagated along the horizontal axis towards the N‐domain with the consequence of pore widening. The N‐terminal network composed of electrostatic interactions between E9, R23 and D27 is clearly visible in our crystal structures. This network connects the N‐domain (residues 1 – 19) to the ClpP core and helps to stabilise the β‐hairpin loops that line the pore (Figures 3 and 4), corroborating previous results which assign a crucial role to these residues in the organisation of the entrance pores in *E. coli* and *N. meningitidis*.^33^ These β‐hairpins and even the extreme N termini of most ClpP subunits are exceptionally well resolved in the presented structures. In the apo structure of *S. aureus* ClpP presented here, two distinct conformations can be differentiated, like in a previous crystal structure of ClpP.^48^ In one conformation, the ordered β‐hairpin loop points upwards, and in the second conformation a structural inversion of the first 19 amino acids destroys the hairpin and allows the entire disordered region to collapse into the pore (Figures 3, 4 and S2). In our apo‐ClpP structure, where two out of seven subunits are in this disordered state, the pore is effectively closed. Such an inversion can also be seen in the apo‐ClpP structures from *Coxiella burnetti* (PDB ID: 3Q7H) and *Francisella tularensis* (PDB: 5G1S and 5G1R).^56^ The first documentation of a so‐called “up‐conformation” and “down‐conformation” in a crystal structure of *E. coli* ClpP^48^ triggered several investigations into the N‐terminal gate.^26^, ^33^, ^47^, ^57^, ^58^ Although the term up conformation is uniformly used to describe the ordered upwards pointing β‐hairpins (with corresponding downwards facing extreme N termini), the down conformation was used to describe a disordered state of which only a snap‐shot had been caught before.^48^, ^57^, ^59^ In this reported state, the N terminus of one in seven ClpP subunits was kinked and the electron density of the first amino acids was found inside the pore.^48^ Based on this *E. coli* ClpP crystal structure which introduced the conundrum of non‐homogeneously structured ClpP N‐termini, a hydrophobic plug that closes the pore and consists of amino acids M1‐V7 was proposed.^48^, ^58^ While we confirm the general concept of pore closure by the N‐terminal domain, the orientation of the N‐termini in the structure presented here differs from that first account of the closed pore. In the current structure, the N termini of two subunits (M1‐V7) are inverted and point upwards with a slight tilt towards the barrel centre and the amino acids E9‐D19 extend into the substrate entry channel contributing to pore blockage. Whether this represents a case specific to ClpPs from certain genera or whether disordered N‐termini can adopt both orientations, is still unknown. The consensus between these structures is an inherent asymmetry within the N‐terminal domains that allows one or more subunits to deviate from a structured hairpin and thereby extend into the channel resulting in complete closure. When ADEP binds, it stabilises the upwards pointing β‐hairpin conformation. As all ClpP subunits within a ring adopt this up conformation, the pore lumen is no longer blocked. In addition, a subtle but marked outwards movement of the entire N‐terminal domain occurs (Figures 3A and S2). These two structural shifts are jointly responsible for the effective increase in pore diameter.

The SaClpP mutant proteins discussed so far demonstrate that ADEP exposure leads to nonfunctional ClpP variants, which are defective in tetradecamer formation and consequently catalysis. In our biochemical analysis, we put a particular focus on *S. aureus*, but ClpP is conserved in Firmicutes (Figure S10), and homologous regions serve the same function. Mutations in the oligomerisation sensor and the ring‐ring interaction network did also occur in other species listed in Table 3. Besides, there are further mutations of the equatorial interphase, where many amino exchanges can be expected to disturb proper tetradecamer formation, and there are also mutations of the catalytic triad. Considering the high‐level resistance that most mutants displayed against ADEP, it is very likely that most (if not all) of those are impaired in the function of ClpP.

However, there are also mutations of the ADEP binding pocket, which have to be considered. The SaClpP M190T mutant protein was not impaired in catalysis. It is a competent tetradecamer and was even slightly more active than wild‐type ClpP in repeated peptide degradation experiments based on different protein preparations (Figure 6). This mutation is located in the ADEP binding site (Figures 5 and S1) and the corresponding mutant protein reacted to ADEP in the same manner as the wild type, although slightly higher compound concentrations were needed for full activation (Figure 6). The mutant was also impaired in its interaction with ClpX (Figure 6), and according to our current knowledge on the conservative binding mode of the other ATPases ClpC and ClpE, they are most probably affected in the same way. Consequently, also the ADEP binding site mutant is impaired with regard to the physiological functions of ClpP. This notion is corroborated by our observation that all five ClpP mutants of *S. aureus* 133, including the ADEP‐binding site mutant, lacked haemolysis (Figure S7) and showed elevated sensitivity to oxidative stress (Figure S8).

This result is an important finding in the context of our second aim, that is, to better understand the implications of ClpP mutants in the light of future ADEP therapy. There is a multitude of publications that report on the central role of ClpP in surviving stress conditions and in regulating bacterial virulence in various Firmicutes species (for reviews, see ref.^14^, ^15^, ^60^). In *S. aureus* the expression of several global virulence regulators from the *sar*/*agr* regulatory network is affected by the absence of ClpP.^15^, ^43^, ^44^ *In vivo*, SaClpP deletion mutants were attenuated in a skin abscess model, during septicaemia and during catheter infections.^43^, ^61^, ^62^ Also, small molecule inhibitors of ClpP reduced the virulence of *S. aureus* in murine infections^40^, ^41^ and the Clp‐ATPase ClpX was likewise validated *in vivo* as an anti‐virulence target for treating *S. aureus* infections.^43^, ^61^, ^63^ Expanding on species, lack of functional ClpP reduced the capacity of *S. epidermidis* to establish a biofilm infection.^64^ ClpP deficient *S. pneumoniae* failed to colonise the lung and led to a considerably higher overall survival rate than the wild type.^65^, ^66^ Among Firmicutes, also in *Listeria monocytogenes* ClpP is required for intracellular survival in macrophages and virulence^67^ and there are also reports on the importance of ClpP for the virulence of Gram‐negative species.^60^

The well‐documented necessity of ClpP for the establishment of virulence in many bacterial species and the finding that the Clp protease system is probably impaired in many (if not all) high‐level ADEP‐resistant mutants implies that the high spontaneous resistance rate observed for ADEP *in vitro* cannot be transferred to the *in vivo* situation. Rather, many ClpP mutants selected by ADEP *in vivo* can be expected to have a strong fitness deficit in the infection process. The whole concept of using ClpP as a broad‐spectrum anti‐virulence target is based on the notion that the lack of functional ClpP will reduce the virulence of the respective pathogen. However, like with all generalisations, care must still be taken. ClpP mutations can be selected under certain conditions *in vivo*, and there is a correlation between the lack of ClpP function and reduced susceptibility to cell wall active antibiotics.^15^ In a set of 39 clinical vancomycin intermediate resistant *S. aureus* (VISA) strains, three ClpP mutations were found (M1V, H83V and R152H).^68^ It is also notable that the role of ClpP for virulence regulation in enterococci is not established. It is promising that in a study on the therapeutic potential of ADEP in enterococci, 73 enterococcal isolates, including vancomycin and multidrug‐resistant strains, showed uniformly low MIC values for ADEP4 (MIC_90_ of 0.031 μg mL^−1^ and 0.063 μg mL^−1^ for *E. faecalis* and *E. faecium*, respectively) ruling out pre‐existing ClpP mutants among the set.^13^

Further studies are warranted to determine to which extent ADEP will select for ClpP mutations *in vivo*, and this aspect will need to be carefully monitored during preclinical and clinical studies. The ClpP mutations that occurred in all ADEP‐resistant mutants across species in this study emphasise that ADEP acts on a single target, and ClpP is encoded by a single gene in most pathogenic species. Thus, combination therapy is probably recommendable, in order to preserve the new mechanism for as long as possible. It is encouraging that in previous studies, ADEP performed well in combination with marked antibiotics from various classes.^11^, ^13^

## Conclusion

In summary, ClpP mutations can be rapidly selected by ADEP exposure *in vitro* and resistance rates in the range of 10^−6^ were determined for all ADEP derivatives and Firmicutes species tested. All ADEP‐resistant mutants analysed carried mutations in ClpP, confirming ClpP as the sole phenotypically relevant target of ADEP, and most mutants were high‐level resistant (MIC >64 μg mL^−1^). The mutated ClpP proteins characterised biochemically in this study were out‐of‐function with severe defects in ring‐ring interaction and consequently catalysis or in the interaction with the Clp‐ATPase ClpX. Given the prominent role of ClpP as a global regulator of bacterial virulence in many pathogens and the out‐of‐function phenotype observed here, it is highly probable that ClpP mutants will be selected by ADEP at a much lower rate *in vivo*. From a mechanistic point of view, we demonstrate a prominent role of A133 and D173 in mediating ring‐ring interactions in *S. aureus ClpP* and confirm the same for Q132, which had been previously described as being part of the oligomerisation sensor. The compilation presented here is the broadest collection of ADEP‐resistant mutants sequenced to date. Based on the fact that selection pressure was towards ClpP out‐of‐function mutations, the compilation points towards residues with important roles and may guide future studies on the operation mode of ClpP. The well‐resolved N‐domains in our crystal structures of *S. aureus* ClpP allow the pore gating mechanism to be followed. In apo‐ClpP residues 1–19 are highly flexible, dynamically alternating between two states. The first state is characterised by an ordered upwards‐pointing β‐hairpin, stabilised by a network of electrostatic interactions between E9, R23 and D27. The second is a state of disorder, here presenting itself as a structural inversion, in which the N‐terminal amino acids block the pore. Upon binding of ADEP, all ClpP subunits within a ring uniformly adopt the β‐hairpin conformation. In addition, an outwards movement of the N‐domain occurs. Together, these two movements are responsible for ADEP‐mediated pore opening.

## Experimental Section

**Determination of antibacterial activity**: ADEP1 was purified from the culture supernatant of *S. hawaiiensis* NRRL 15010 as described.^4^ ADEPs 2, 4, 7, 9 were custom synthesised by EMC Microcollections, Tübingen, or provided by AiCuris, Wuppertal, Germany. The minimal inhibitory concentration (MIC) was determined by broth microdilution in Mueller‐Hinton broth (without cation adjustment) according to the guidelines of the Clinical Laboratory Standards Institute (CLSI).^69^ The inoculum was set to 5×10^4^ CFU mL^−1^ to allow some distance to the determined spontaneous resistance frequencies. ADEP1 was freshly dissolved in DMSO for the MIC determinations, as it is rather unstable. All other ADEPs were used from DMSO stock solutions kept at −20 °C.

**Screening for ADEP‐resistant mutants and determination of resistance rates**: Single colonies of wild‐type strains grown on Mueller‐Hinton agar plates overnight were suspended in sterile saline and a final inoculum of 5×10^6^ cells was spread on plates supplemented with different ADEP congeners at 4× and 10× the MIC. For *B. subtilis* strain 3x*clpP*, ADEP7‐containing Mueller‐Hinton agar plates were additionally supplemented with 1 mM IPTG. Resistant colonies were counted after 24 h at 37 °C and colonies were randomly chosen for MIC determinations and sequencing. Mutations in the *clpP* gene of the respective isolated mutants were analysed by amplifying the *clpP* gene (and upstream promoter region) by PCR and sequencing of the resulting PCR amplicons (LGC genomics). Sequencing data were analysed by using SnapGene software (GSL Biotech).

**Construction of plasmids and bacterial strains**: All bacterial strains, plasmids and oligonucleotides used in this study are listed in Table S1. Standard techniques were employed for all cloning experiments.^70^ To introduce point mutations into *clpP* genes from *S. aureus* or *B. subtilis*, the QuikChange II site‐directed mutagenesis kit (Agilent Technologies) was employed using the plasmid pET301_*SaclpP* and pClpP11, respectively, as templates. For the construction of plasmids pAPNC_*Pspac*_*clpP* and pBS2E_*Pspac*_*clpP*, full‐length *clpP* amplicons, PCR‐amplified from genomic DNA of *B. subtilis* 168, were inserted into the vector backbones (linearised by PCR) by the Gibson isothermal assembly method (Gibson assembly Master Mix, NEB). The resulting plasmids were transformed into *B. subtilis* 168 for ectopic integration at the *lacA* and *aprE* loci, respectively, to yield strain 3x*clpP*. For construction of plasmids pET22b_*BsclpP*‐*his* and pET22b_*BsclpP*_A133S‐*his*, *clpP* was amplified from plasmids pClpP11 and pClpP11(g397t), respectively. The resulting PCR fragments were digested with NdeI and XhoI and ligated into plasmid pET22b, which had previously been digested with the same restriction enzymes. Construction of plasmid pET11a_*clpP*‐strep was performed by employing Gibson isothermal assembly*. E. coli* XL‐10 Gold (Agilent Technologies) and *E. coli* JM109 were used as cloning hosts for plasmid construction.

**Protein purification**: ClpP proteins from *S. aureus* and *B. subtilis* were expressed in SG1146a (Δ*clpP*) cells harbouring the respective expression plasmid (Table S1). SaClpP protein variants (without affinity tag) were purified on an ÄKTA Pure system equipped with a HiTrap Q XL column using buffer A (20 mM Tris‐HCl pH 8) and buffer B (20 mM Tris‐HCl pH 8, 1 M NaCl). Elution was carried out in a step‐wise gradient with the desired protein usually eluting at 30 % buffer B. Eluted samples were applied to a Superdex 200 HiLoad 16/600 preparation grade size‐exclusion column and eluted with an isocratic gradient of storage buffer (20 mM Tris‐HCl pH 7, 100 mM NaCl, 10 % *v/v* glycerol). For purification of C‐terminally His_6_‐tagged BsClpP variants, cells were grown in lysogeny broth to an OD_600_ of about 0.5, protein expression was induced by 1 mM IPTG for 4 h, followed by cell harvest and lysis (PreCellys homogeniser). Protein purification was conducted by nickel‐nitrilotriacetic acid affinity chromatography. For purification of C‐terminally strep‐tagged BsClpP, cells were resuspended in ice‐cold lysis/wash buffer (100 mM Tris‐HCl pH 8, 150 mM NaCl, 1 mM EDTA) and lysates were prepared as described above. Protein purification was conducted employing StrepTrap HP 1 mL columns and elution was carried out in lysis/wash buffer with 2.5 mM d‐desthiobiotin added (ÄKTA Start system). Collected samples were further purified by gel filtration in storage buffer. Expression and purification of enhanced GFP carrying a C‐terminal ssrA‐tag for ClpXP degradation assays and of N‐terminally His‐tagged SaClpX were conducted as described.^29^ For protein quantification, the Bradford assay and a Nanodrop spectrophotometer were used and protein quality was analysed by SDS‐PAGE.

**Peptide and protein degradation assays**: Degradation assays with the fluorogenic peptide substrate Suc‐Leu‐Tyr‐7‐amino‐4‐methylcoumarin (Suc‐LY‐AMC) were performed in either BsClpP (50 mM Tris‐HCl, 25 mM MgCl_2_, 200 mM KCl, 2 mM DTT, pH 8) or SaClpP activity buffer (100 mM HEPES pH 7, 100 mM NaCl) in black, flat‐bottom‐96‐well plates (Sarstedt) with a total reaction volume of 100 μl and reaction temperatures of 32 °C and 37 °C for SaClpP and BsClpP, respectively. Purified ClpP proteins (final concentrations as indicated in the respective experiments) and ADEP2 (applied in 1 μl from a DMSO stock solution) were mixed and pre‐incubated for 15 min at reaction temperatures. To start the reaction, Suc‐LY‐AMC (final concentration: 100 μM) in the respective activity buffer was added. Fluorophore release upon peptide hydrolysis was read out in a Tecan Infinite M200Pro plate reader (excitation/emission: 380/460 nm) every 30–60 s for 1 h. Degradation assays with the fluorogenic model protein substrate casein (FITC‐casein, Sigma) were carried out in SaClpP activity buffer in aforementioned plates in a total volume of 100 μl. SaClpP (1 μM) and ADEP2 (concentrations as indicated) were pre‐incubated at 32 °C for 15 min before initiating the reaction by addition of FITC – casein (20 μM final concentration) and fluorescence readout (excitation/emission: 485/535 nm). In both assays, enzyme velocity was determined by linear regression of the initial segment of the fluorescence‐time plot in GraphPad Prism 5. For degradation of GFP‐ssrA, a mixture of SaClpP (2.8 μM), SaClpX (2.4 μM), GFP‐ssrA (0.36 μM) and an artificial ATP regeneration system (4 mM ATP, 20 U mL^−1^ creatine phosphokinase and 16 mM creatine phosphate) were incubated in buffer PZ (25 mM HEPES pH 7.6, 200 mM KCl, 5 mM MgCl_2_, 1 mM DTT, 10 % *v/v* glycerol) in a total reaction volume of 100 μL. After pre‐incubation for 10 min at the reaction temperature of 30 °C, the reaction was started by adding GFP‐ssrA. Decrease of fluorescence was monitored at an emission wavelength of 535 nm in white, flat‐bottom 96‐well‐plates (Greiner). All kinetic assays were performed in triplicates and repeated at least three times. *In vitro* degradation assays of FtsZ were performed with SaClpP in SaClpP activity buffer at 37°C in the presence of ADEP2 at indicated concentrations. Samples were taken at time points indicated and analysed *via* SDS‐PAGE.

### Analytical size‐exclusion chromatography and native PAGE analysis

For analytical size‐exclusion chromatography, an ÄKTA Pure chromatography system equipped with a Superdex 200 3.2 Increase column was employed. When appropriate, protein samples were pre‐incubated with ADEP2 for 30 min at RT. Elution was carried out with an isocratic gradient in SaClpP activity buffer (100 mM HEPES pH 7, 100 mM NaCl).

For analysis by native PAGE, ratios of BsClpP variants in BsClpP activity buffer (final concentrations indicated in the respective figure) were mixed 1 : 1 with native sample buffer (Serva) and subsequently loaded onto Novex Tris‐glycine ready gels (Thermo). Electrophoresis was carried out at 18 V for approx. 16 h in an ice‐cooled chamber followed by staining with either InstantBlue Coomassie protein stain (Sigma) or immunoblotting using mouse‐anti‐Strep (IBA, 1:2000) or mouse anti‐His (IBA, 1:2000) antibodies as described previously.^36^ Rabbit‐anti‐mouse antibodies (IBA, 1:2000) were used as secondary antibodies.

**Crystallisation and structure determination**: After gel filtration, SaClpP was stored in 20 mM Tris‐HCl, pH 7.0 and 100 mM NaCl and concentrated to 11 mg mL^−1^. Both apo and ADEP4‐bound SaClpP were crystallised by the sitting drop vapor diffusion technique at 20 °C. Crystals of the apo protein grew in drops of 0.4 μL volume containing equal amounts of protein and 0.1 M MES sodium salt, pH 6.5 with 0.2 M magnesium acetate and 15 % MPD as a reservoir solution. Mother liquor was used as cryoprotectant prior to vitrification of the crystals in liquid nitrogen at 100 K. For co‐crystallisation, a 2‐fold molar excess of ADEP4 was mixed with SaClpP and incubated for 15 min at 25 °C. SaClpP:ADEP4 crystallised in drops of 0.6 μL volume with protein and reservoir solution (3.5 M sodium formate, pH 7.5) at a ratio of 2 : 1. Crystals were cryoprotected by supplying the mother liquor with 20 % ethylene glycol.

X‐ray diffraction data were recorded at the beamline X06SA, Swiss Light Source (SLS), Villingen, Switzerland, using synchrotron radiation of λ=1.0 Å. They were scaled, indexed and integrated with the program package XDS (Table S3).^71^ The apo protein crystallised in space group P1 with one tetradecamer per asymmetric unit (AU), whilst ADEP4‐bound SaClpP formed crystals in space group P6_5_22 with one heptameric ring in the AU. Both structures were solved by molecular replacement with PHASER^72^ using the extended form of apo SaClpP (PDB: 3V5E)^21^ as search model (the first twenty amino acids of each subunit have been excluded). Cyclic refinement and model building steps were performed using REFMAC5^73^ and COOT^74^, thereby exploiting the seven‐ or 14‐fold noncrystallographic symmetry within the AU, respectively. With one ADEP4 molecule bound to each subunit, clear electron density for the ligand was seen in the SaClpP:ADEP4 structure. The small‐molecule's structure was modelled and minimised with the SYBIL software package. Due to large deviations, the N‐terminal region of all subunits in both structures were built individually.

Final TLS refinement resulted in good values for *R*_work_, *R*_free_ and r.m.s.d. bond and angle values (Table S3). Structure factors and coordinates for both apo and ADEP4‐bound SaClpP were deposited in the RCSB protein data bank and can be accessed via 6TTY and 6TTZ.

## Conflict of interest

The authors declare no conflict of interest.

## Supporting information

As a service to our authors and readers, this journal provides supporting information supplied by the authors. Such materials are peer reviewed and may be re‐organized for online delivery, but are not copy‐edited or typeset. Technical support issues arising from supporting information (other than missing files) should be addressed to the authors.

Supplementary
