# Supplementary material for: Functional Characterisation of ClpP Mutations Conferring Resistance to Acyldepsipeptide Antibiotics in Firmicutes
Source: Chembiochem. 2020 Apr 9;21(14):1997–2012. doi: 10.1002/cbic.201900787 (PMC7496096; doi:10.1002/cbic.201900787)
Supplement: Supplementary file 1 — Supplementary [file CBIC-21-1997-s001.pdf]

ChemBioChem

Supporting Information

Functional Characterisation of ClpP Mutations Conferring Resistance to Acyldepsipeptide Antibiotics in Firmicutes

Imran T. Malik,* Rebeca Pereira, Marie-Theres Vielberg, Christian Mayer, Jan Straetener, Dhana Thomy, Kirsten Famulla, Helena Castro, Peter Sass, Michael Groll, and Heike Brötz-Oesterhelt*© 2020 The Authors. Published by Wiley-VCH Verlag GmbH & Co. KGaA.
This is an open access article under the terms of the Creative Commons Attribution License, which permits use, distribution and reproduction in any medium, provided the original work is properly cited.This article is part of a Special Collection on Microbial Biosynthesis and Interactions. To view the complete collection, visit our

Table of contents:

I. Supporting Figures **S2**
FigS1: ADEP binding site S2
FigS2: SaClpP crystal structure S3
FigS3: SaClpP crystal structure: Detailed view of D27 residue S3
FigS4: Biochemical analysis of N-terminal gate amino acid residues S5
FigS5: Processivity of β-casein degradation by *S. aureus* ClpP S6
FigS6: Gel filtration analysis of *S. aureus* ClpP mutants S7
FigS7: Hemolysis assay with *S. aureus* 133 RC1 – RC5 .... S8
FigS8: Hydrogen peroxide assay with *S. aureus* 133 RC1 – RC5 .... S9
FigS9: BsClpP expression analysis and control experiments S10
FigS10: Multiple sequence alignment of ClpPs from examined Firmicutes S11
II. Supporting Tables **S12**
TableS1: Strains, plasmids and oligonucleotides used in this study S12
TableS2: Overview of *S. aureus* ClpP mutant biochemistry S14
TableS3: Crystallization data collection and refinement statistics S14
III. Supporting References **S15**

I. Supporting Figures

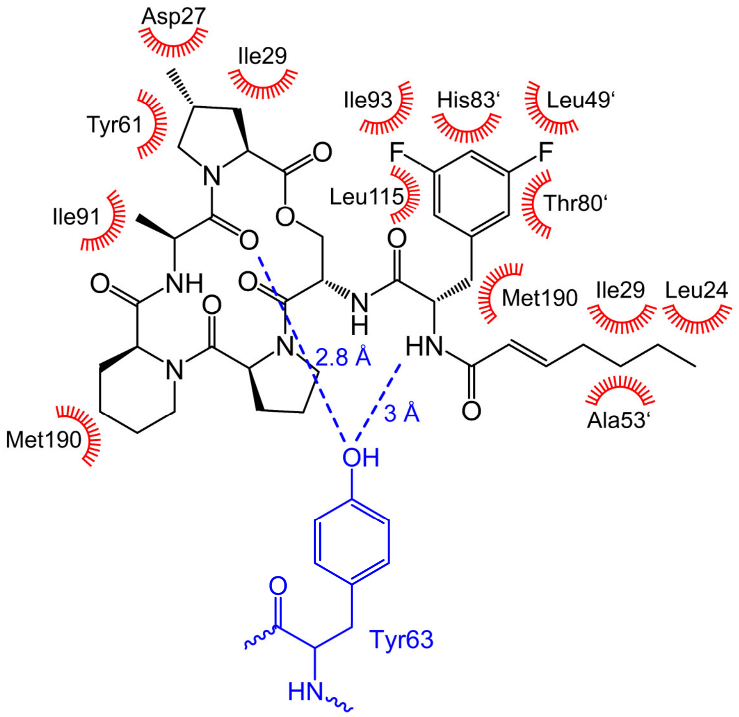

Figure S1. Schematic showing interactions between ADEP4 and SaClpP. The hydrogen bonds between ADEP4 and Tyr63 are denoted as blue dotted lines, hydrophobic interactions between the ADEP4 molecule and amino acid residues of two ClpP subunits are marked as red starbursts.

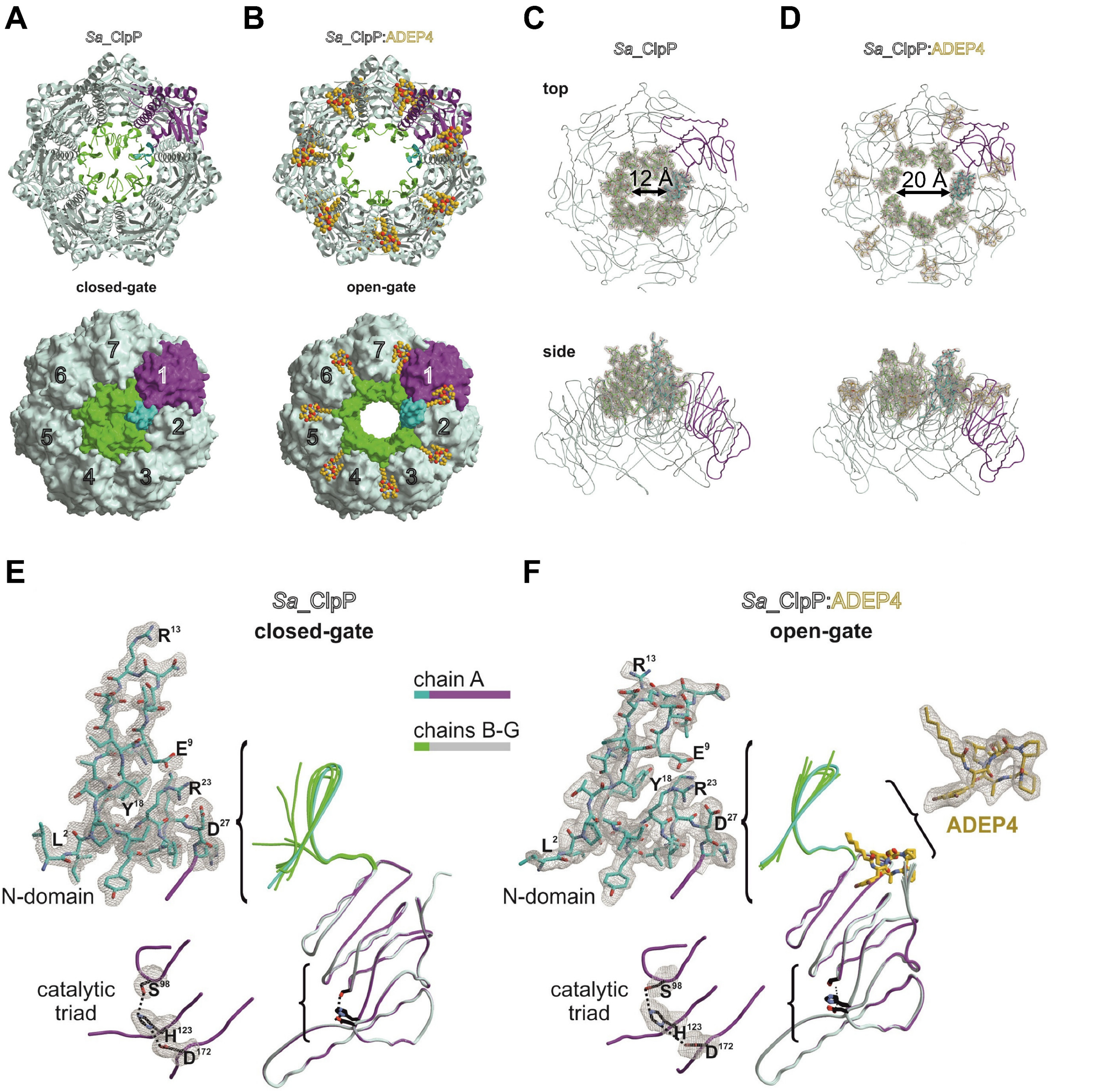

Figure S2. Crystal structures of SaClpP in A) *apo* (closed conformation, PDB ID 6TTY) and B) in complex with the acyldepsipeptide antibiotic ADEP4 (open conformation, PDB ID 6TTZ). The upper panels illustrate ribbon representations of the protease along the 7-fold symmetry axis (top view). The domains of one subunit are colored in cyan (amino acids 1-27) and magenta (amino acids 27-195). All remaining subunits are depicted in green, and grey, respectively. The activator ADEP4 is shown as balls (carbon atoms: gold, nitrogen atoms: blue, oxygen atoms: red). Strands β1, β2 and helix α1 of the N-domains form the entrance gate into ClpP, while the proteolytic active sites are sequestered in the central hydrolytic chamber formed by the C-domains. The lower panels depict surface representations of the SaClpP and SaClpP:ADEP structures. Subunits are numbered from 1-7.
$2F_O$-F_C electron density maps of the SaClpP protease in its closed C) and open D) conformation. The respective electron densities are shown in grey for the N-terminal domains and ADEP4 (contour level to 1σ). Colour coding is according to panels A) and B). The upper panels illustrate the top and the lower panels the side view of one SaClpP ring, respectively. The N-domains exclusively adopt a β-hairpin conformation in the SaClpP:ADEP4 structure, whereas two out of seven subunits in the *apo*-structure display an inverted and disordered orientation. The distance between two properly folded β-hairpins in the *apo*-SaClpP amounts to roughly 12 angstroms (disregarding pore closure by inverted N-domains). The pore diameter in the SaClpP:ADEP4 structure increases to 20 angstroms illustrating an outward movement of all of the N-terminal domains in the presence of ADEP4.
Detailed view of the N-terminal domains of SaClpP in E) *apo* and in F) complex with ADEP4 represented by an overlay of seven ClpP subunits. Special emphasis lies on the electron density maps of residues 1 – 27 and the catalytic triad. Colour coding is equivalent to panel A and B. Both structures show exceptionally high-resolution details about the N-terminal domains.

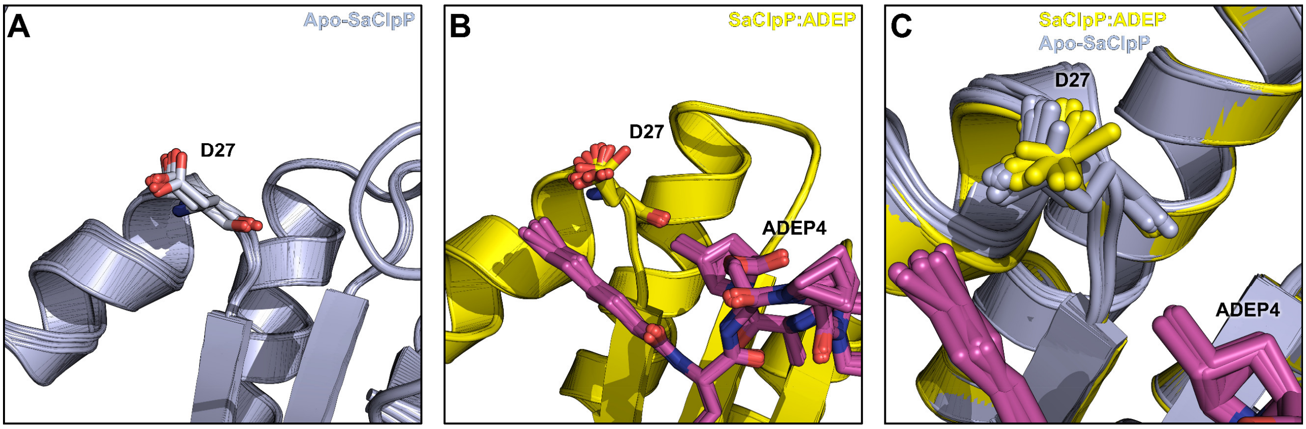

Figure S3. Overlay of the D27 moieties of seven SaClpP subunits in A) *apo* and B) in complex with ADEP4. C) Overlay of the subunits depicted in A) and B) shows a slight positional shift of the D27 residue in the SaClpP:ADEP structure probably because of repulsion by the hydrophobic tail region of ADEP4. The individual D27 side-chains display different degrees of rotation.

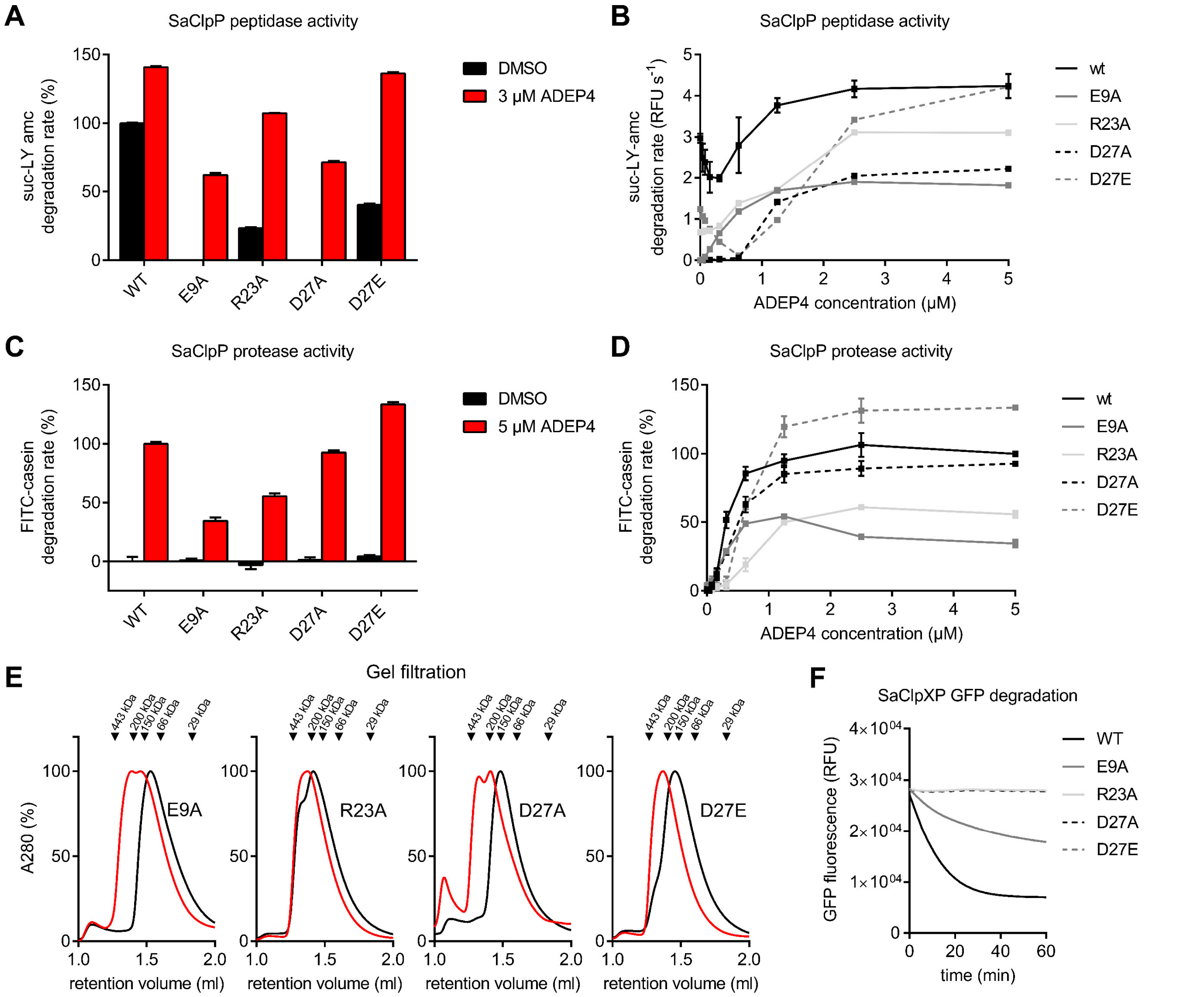

Figure S4. Biochemical characterization of the amino acid residues involved in the N-terminal electrostatic network of SaClpP. The N-domain mutants E9, R23 and D27A were generated, purified, and tested for *in vitro* activity and oligomeric state. For a more detailed characterization of the critical D27 residue, which undergoes major structural shifts in response to ADEP binding, we additionally introduced the homologous glutamate residue of *E. coli* ClpP at this position (D27E). A) Suc-LY-AMC degradation by SaClpP as a measure of catalytic function. All mutants showed defects in intrinsic catalytic activity (E9A and D27A lose all activity) but could be activated by the addition of ADEP4 to varying degrees. These defects in catalysis can be primarily attributed to a loss of oligomeric integrity (see panel E). Error bars indicate S.D. One representative of three independent experiments is shown. B) Suc-LY-AMC degradation over an ADEP4 concentration range from 0 – 5 µM. The inhibition of catalysis rate at low ADEP concentrations is characteristic for intrinsically tetradecameric SaClpP as well as tetradecameric preparations of BsClpP[1]. Among the set of mutants depicted in this figure, it could only be observed for the SaClpP WT and the D27E mutant, which is reflective of the wildtype situation in *E. coli* ClpP. Error bars indicate S.D. One representative of three independent experiments is shown. C) FITC-casein degradation by the N-terminal network mutants as a measure of pore opening function of ADEP4. When exposed to 5 µM of ADEP4, all mutants retained the ability to degrade FITC-casein with the D27E mutant even exceeding WT levels. Error bars indicate S.D. One representative of three independent experiments is shown. D) FITC-casein degradation over an ADEP4 concentration range from 0 – 5 µM. R23A displayed a severe loss of ADEP4 binding affinity characterized by its delay in response to low ADEP4 concentrations. Error bars indicate S.D. One representative of three independent experiments is shown. E) Analysis of the oligomeric state of the N-terminal network mutants via gel filtration in the absence (black lines) and presence (red lines) of 150 µM of ADEP4. The D27E mutant is the only one among the set of N-terminal network mutants that showed a residual tetradecameric fraction in the absence of ADEP4 (note the shoulder on the left in the black curve). When exposed to ADEP4, it was transformed into a tetradecamer which corresponds to a molecular weight of roughly 301 kDa. The alanine substitution mutants E9A and D27A could only partially achieve tetradecameric conformation in the presence of ADEP4 with almost equal amounts remaining in a heptameric state (roughly 150.5 kDa). The R23A mutant displayed a non-homogenous tetradecameric fraction. One representative of three independent experiments is shown. F) GFP-ssrA degradation by SaClpX mixed with stoichiometric amounts of SaClpP. Interestingly, only the E9A mutant retained the ability to functionally pair with SaClpX and degrade GFP, albeit to a lower degree than the SaClpP WT. This finding suggests an important role of the N-terminal domain in the communication between SaClpP and SaClpX. One representative of three independent experiments is shown.

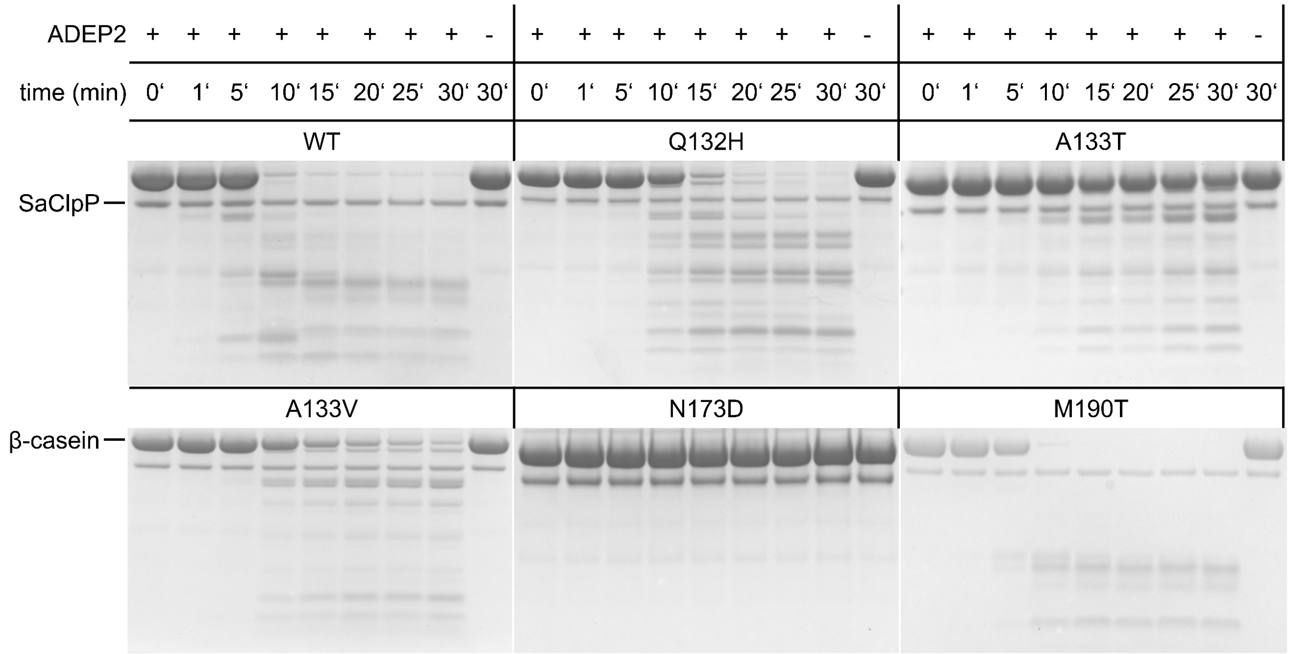

Figure S5. β-casein degradation of ADEP resistant *S. aureus* 133 ClpP (RC1 - 5) mutant proteins in the presence of ADEP2 analyzed by SDS-PAGE. WT and M190T degrade β-casein in the presence of ADEP2 at similar velocities and display similar patterns of degradation bands suggesting similar processivity. The A133V and Q132H mutants, while still being activated by ADEP2, show accumulation of higher molecular weight degradation bands of β-casein indicating decreased processivity. The A133T is strongly impaired in β-casein degradation, the N173D mutant is completely inactive.

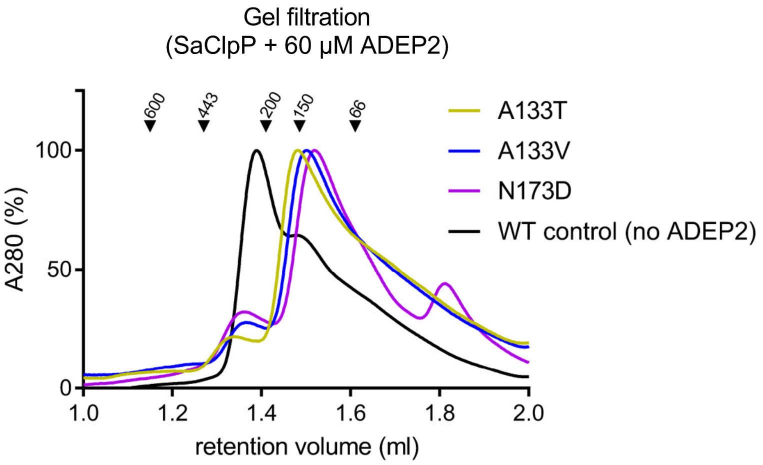

Figure S6. Gel filtration of ADEP-resistant *S. aureus* 133 ClpP mutant proteins at 60 µM of ADEP2. Mutants A133T, A133V, and N173D, which failed to adopt a tetradecameric state at 30 µM, could be partially transformed into tetradecamers at the higher ADEP2 concentration (see small maxima at roughly 300 kDa).

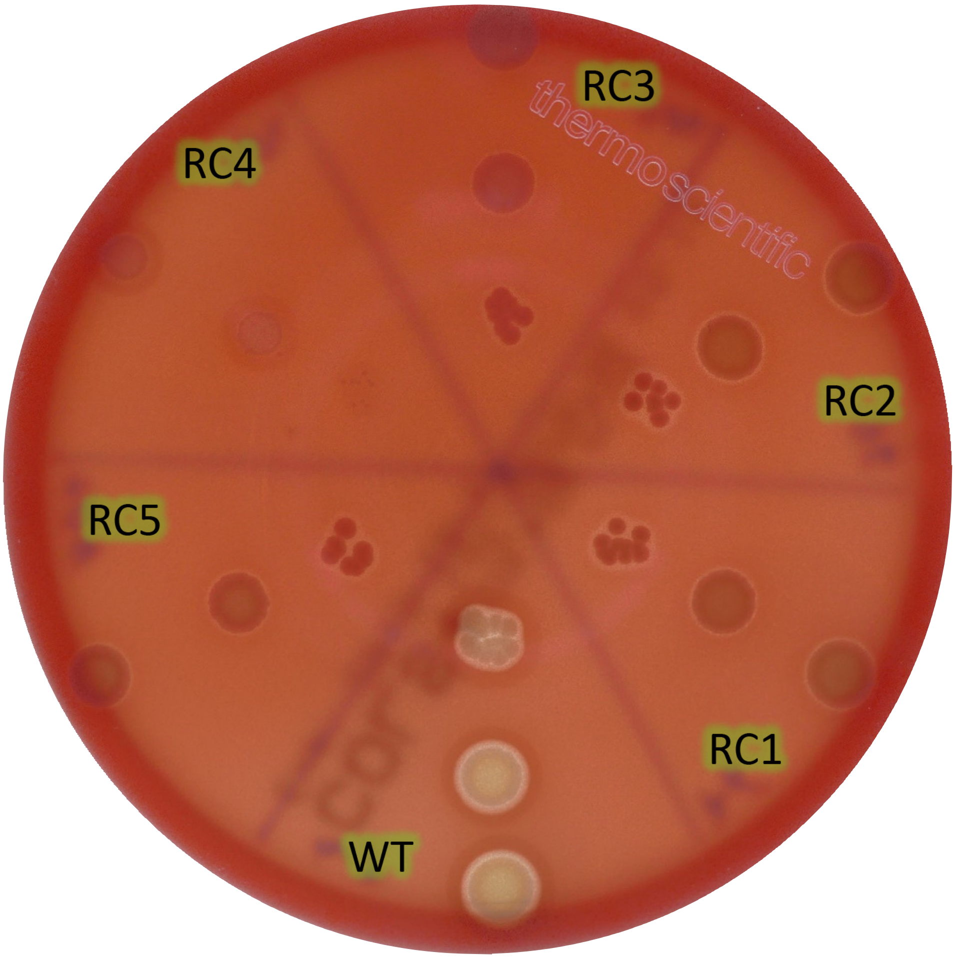

Figure S7. All five ADEP-resistant mutants that were characterised in detail in this study are impaired in hemolysis. Wildtype *S. aureus* 133 and its 5 ADEP-resistant derivatives RC 1 to 5 were grown overnight on cation-adjusted MH agar and resuspended in fresh MH (without cation adjustment) to an OD_{600} of 0.1. Then 1 µl of this suspension corresponding to 1.5×10^5 colony forming units (CFU) was applied to a Columbia blood agar plate (outer spot). Then the suspension was diluted 1:100 and 1:10000 to yield 1.5×10^3 CFU and 1.5×10^1 CFU in the middle and innermost spot, respectively. Plates were incubated at 37°C overnight.

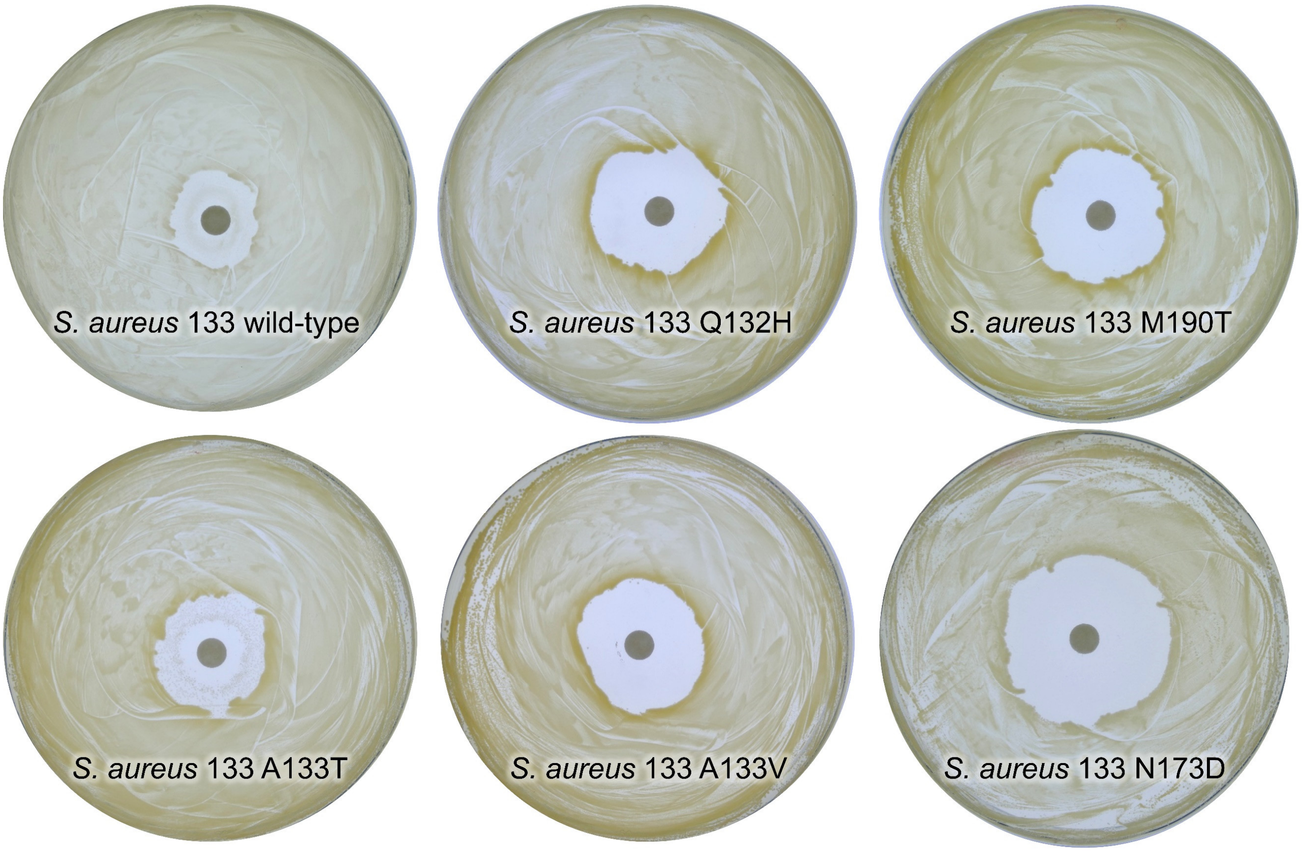

Figure S8. The ADEP-resistant *S. aureus* mutants characterised in detail in this study show increased susceptibility to hydrogen peroxide. 50 µl of stationary phase cultures were plated on TSB and 10 µl of a hydrogen peroxide solution (3%) were spotted on discs in the middle of the plates. Plates were incubated at 37 °C overnight.

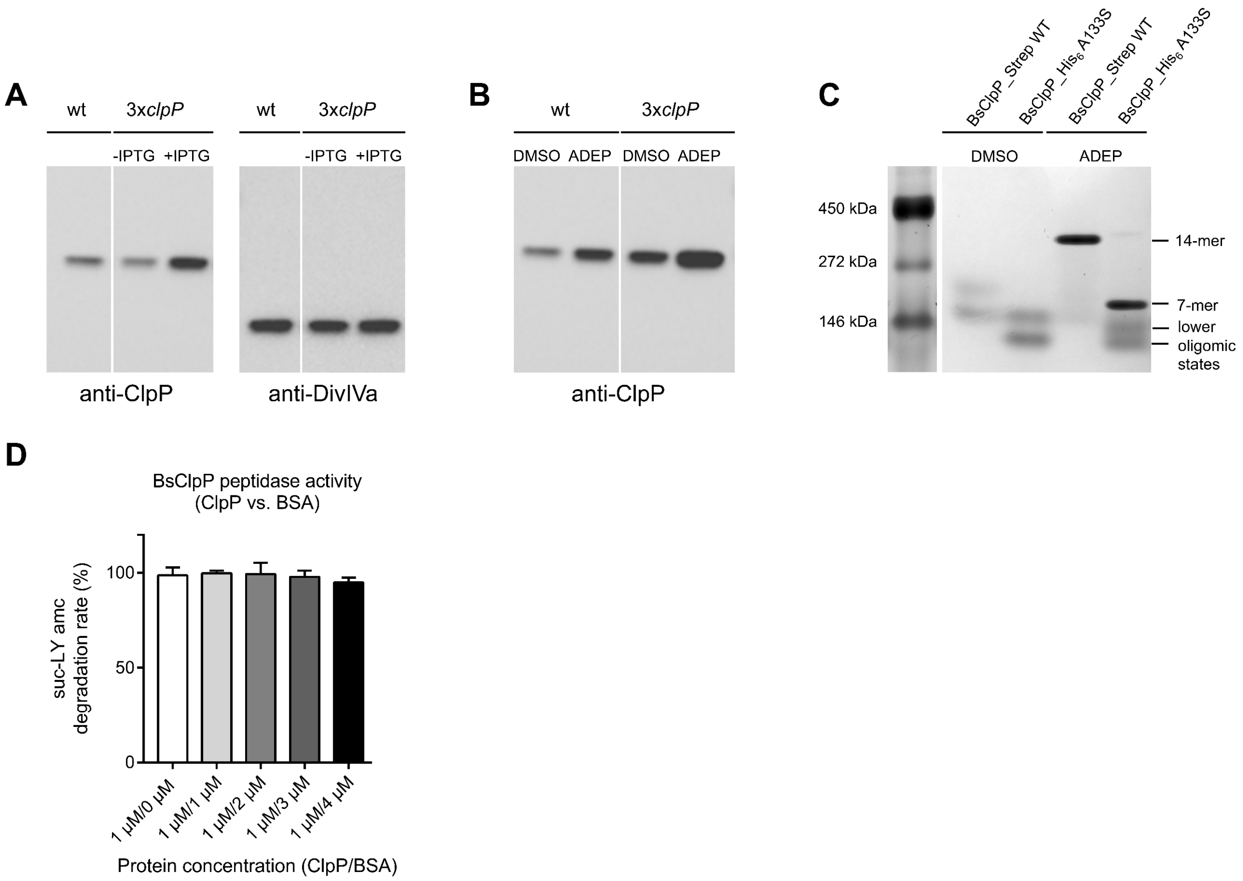

Figure S9. Expression level of ClpP in *B. subtilis* (A,B) and control experiments for isolated BsClpP proteins (C,D). A) ClpP expression in the absence of ADEP. Exponentially growing *B. subtilis* 168 wildtype (containing only 1 *clpP* copy behind its native promoter) and its genetically engineered derivative *B. subtilis* 3xclpP (containing two inducible ectopic *clpP* copies plus the native copy behind the native promoter) were analysed for their expression level of the ClpP protein. Both strains were grown overnight at 37° C in LB medium, diluted in fresh LB (with or without 1mM IPTG) to an OD600 of 0.05 and grown to approx. OD 0.4. Cells were harvested, resuspended in lysis buffer (300 mM NaCl, 50 mM NaH2PO4, pH 8), supplemented with EDTA-free mini protease inhibitor (Roche) and disrupted in a PreCellys homogenizer. Protein concentrations of clear lysates were adjusted and 25 µg of total soluble protein per lane were separated by SDS-PAGE. Proteins were analysed by immunoblotting using rabbit-anti-ClpP (1:10.000)[2] and rabbit-anti-DivIVa (1:5000) antibodies and subjected to chemiluminescence detection (ChemiDoc documentation system, Biorad). DivIVA was used as an unrelated loading control and detected by anti-DivIVA. Images are representative of three biological replicates. B) Effect of ADEP on ClpP expression. The experiment was performed as described above. When *B. subtilis* WT and IPTG-induced *B. subtilis* 3xclpP cultures reached an OD600 of 0.2, they were treated with ADEP2 (0.06 µg ml-1) for 30 min (until they reached OD 0.4). In both strains, ClpP levels were strongly increased by ADEP. DMSO was added to samples that did not contain ADEP. Images are representative of three biological replicates. C) Impact of ADEP on the oligomeric states of the purified proteins BsClpP WT (carrying a C-terminal Step-tag) and BsClpP_A133S (carrying a C-terminal His-Tag). After purification, wildtype BsClpP is not a tetradecamer. Native PAGE analysis shows that upon ADEP addition BsClpP WT appears uniformly in a higher molecular weight band of approx. 320 kDa indicating tetradecamer formation. In contrast, the A133S mutant predominantly shows heptamers in the presence of ADEP. In a control DMSO failed to induce 14-mers. Images are representative of three biological replicates. D) Peptidase activity of BsClpP WT in the presence of rising amounts of bovine serum albumin (BSA). BSA did not affect the peptidase activity of ADEP-activated BsClpP. The concentration of BsClpP WT was kept at 1 µM final concentration in all samples and mixed with increasing concentrations of BSA. ADEP2 was added at a concentration of 10 µM. Error bars indicate S.D. (three independent experiments).

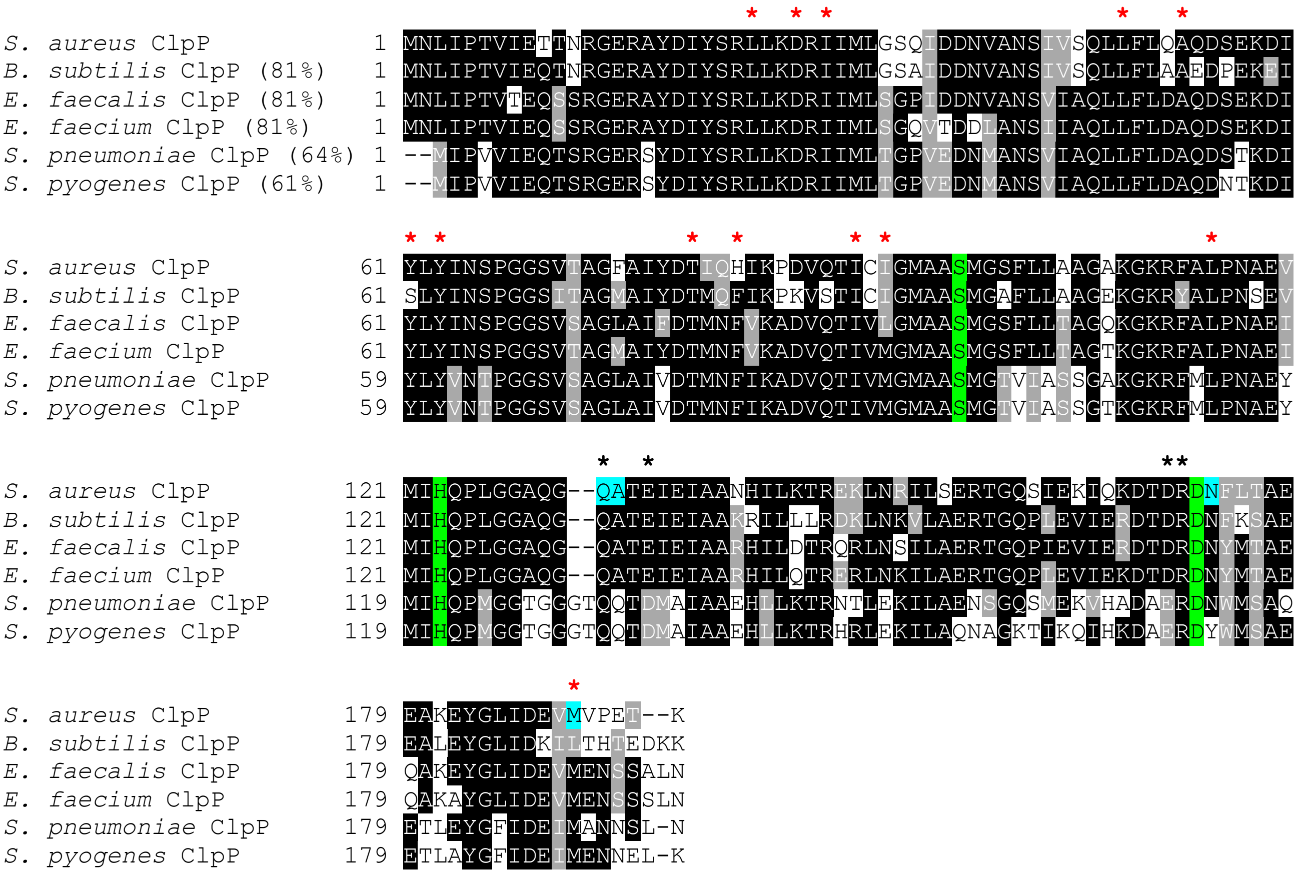

Figure S10. Multiple amino acid sequence alignment of ClpPs from the Firmicutes examined in this study. *S. aureus* ClpP (NCBI: OBY01723) shares very high sequence identities with ClpPs from *B. subtilis* (NCBI: PSL99305), *E. faecalis* (NCBI: WP_010825783), *E. faecium* (NCBI: AUC72190), and high sequence identities with *S. pneumoniae* ClpP (NCBI: AVN85794) and *S. pyogenes* ClpP (NCBI: ANC26642). Active site amino acids are highlighted in light green, amino acids involved in resistance of *S. aureus* 133 resistant clones RC1-5 are highlighted in light blue. Black asterisks on the top denote oligomerization sensor residues and red asterisks mark amino acid residues comprising the ADEP binding site as identified with the help of the SaClpP:ADEP4 crystal structure in this study. Alignments were generated with MAFFT[3] and analysed with BOXSHADE. Sequence identities were determined by protein blast and are stated in parenthesis.

II. Supporting Tables

Table S1. Strains, plasmids and oligonucleotides used in this study

Strain/plasmid	Genotype/relevant characteristic(s)	Reference/source
B. subtilis		
168	*trpC2*; wild type strain	ref[4]
3xclpP	*trpC2 lacA::(Pspac-clpP* erm), *aprE::(Pspac-clpP cat)*	this study
E. faecalis		
27159	clinical isolate	AiCuris
E. faecium		
L4001	clinical isolate	AiCuris
S. aureus		
ATCC 29213	clinical isolate, reference strain CLSI	ref[5]
133	clinical isolate	AiCuris
Newman	clinical isolate, ATCC25904, $rsbU^+$	D. Frees[6]
Newman Δ*clpP*	clinical isolate, ATCC25904, $rsbU^+$, $clpP^-$	D. Frees[7]
JE-2	clinical isolate, community-acquired MRSA USA300	ref[8]
JE-2 *clpP*::*tnerm*	clinical isolate, ca-MRSA USA300, *clpP*::*tnerm*	Nebraska transposon library[8]
NCTC 8325-4	*(8325 derivative lacking prophages)* $rbsU^-$, hib^-	D. Frees[9]
NCTC 8325-4 Δ*clpP*	*(8325 lacking prophages)* $rbsU^-$, hib^-, $clpP^-$	D. Frees[10]
S. pneumoniae		
G9a	clinical isolate	AiCuris
S. pyogenes		
Wacker	clinical isolate	AiCuris
E. coli		
JM109	*endA1 glnV44 thi-1 relA1 gyrA96 recA1* $mcrB^+$ *Δ(lac-proAB) e14- [F' traD36* $proAB^+$ $lacI^q$ *lacZΔM15] hsdR17*($r_K^-m_K^+$), subcloning host	laboratory stock
XL10-Gold	*TetrD(mcrA)183 D(mcrCB-hsdSMR-mrr)173 endA1 supE44 thi-1 recA1 gyrA96 relA1 lac Hte [F´ proAB lacIqZDM15 Tn10(Tetr) Amy* Cam^R*]*, subcloning host	Agilent Technologies
SG1146a	*clpP::cat*, lon^-, λDE3 lysogen; BL21 derivative; expression host	S. Gottesman[11,12]
**Plasmids**		
pET301_SaclpP	pET301 expressing vector + tag-free coding sequence of *S. aureus* NCTC 8235 *clpP*	S. Sieber[13]
pET301_SaclpP_Q132H	pET301_SaclpP carrying a point mutation at position 396 (A→T) in the *S. aureus* NCTC 8235 *clpP* gene	this study
pET301_SaclpP_A133T	pET301_SaclpP carrying a point mutation at position 397 (G→A) in the *S. aureus* NCTC 8235 *clpP* gene	this study
pET301_SaclpP_A133V	pET301_SaclpP carrying a point mutation at position 398 (C→T) in the *S. aureus* NCTC 8235 *clpP* gene	this study
pET301_SaclpP_N173D	pET301_SaclpP carrying a point mutation at position 517 (A→G) in the *S. aureus* NCTC 8235 *clpP* gene	this study
pET301_SaclpP_M190T	pET301_SaclpP carrying a point mutation at position 569 (T→C) in the *S. aureus* NCTC 8235 *clpP* gene	this study
pClpP11	expression vector pQE70 (Qiagen) + ORF BSU34540 (*B. subtilis clpP*) with C-terminal His_6-tag	ref[14]
pClpP11_A133S	pClpP11 carrying a transversion (G→T) at position 397 in the *B. subtilis clpP* gene	this study
pET22b	expression vector carrying a C-terminal His_6-tag sequence	Novagen
pET22b_BsclpP-his	pET22b + ORF BSU34540 (*B. subtilis clpP*)	this study
pET22b_BsclpP_A133S-his	pET22b_*clpP-his* carrying a transversion (G→T) at position 397 in the *B. subtilis clpP* gene	this study
pAPNC	empty vector, *Pspac*, integration at *aprE, bla cat*	L. Hamoen[15,16]
pAPNC_ BsclpP	integration at *aprE, Pspac-BsclpP, bla cat*	this study
pBS2E	empty vector, integration at *lacA*, *bla erm*	T. Mascher[17], (Addgene plasmid #55169; http://n2t.net/addgene:55169; RRID: Addgene_55169).
pBS2E_ BsclpP	integration at *lacA*, *Pspac-BsclpP*, *bla erm*	this study
pET11a-strep	expression vector pET11a carrying a C-terminal Strep-tag sequence	S. Pan (unpublished)
pET11a_BsclpP-strep	pET11a-strep + ORF BSU34540 (*B. subtilis clpP*)	this study

Oligonucleotides for cloning experiments

Template	Oligonucleotide sequence (5`→3`)	Resulting plasmid
pET301_SaclpP (SDM)	Fw: GTGGTGCTCAAGGACATGCAACTGAAATCG<br>Rv: CGATTTCAGTTGCATGTCCTTGAGCACCAC	pET301_SaclpP_Q132H
pET301_SaclpP (SDM)	Fw: GGCTTAATTGATGAAGTGACGGTACCTGAAAC<br>Rv: GTTTCAGGTACCGTCACTTCATCAATTAAGCC	pET301_SaclpP_M190T
pET301_SaclpP (SDM)	Fw: GGTGGTGCTCAAGGACAAACAACTGAAATCG<br>Rv: CGATTTCAGTTGTTTGTCCTTGAGCACCACC	pET301_SaclpP_A133T
pET301_SaclpP (SDM)	Fw: GACACAGATCGTGATGACTTCTTAACTGCAGAAG<br>Rv: CTTCTGCAGTTAAGAAGTCATCACGATCTGTGTC	pET301_SaclpP_N173D
pET301_SaclpP (SDM)	Fw: GGTGGTGCTCAAGGACAAGTAACTGAAATCG<br>Rv: CGATTTCAGTTACTTGTCCTTGAGCACCACC	pET301_SaclpP_A133V
pET301_SaclpP (SDM)	Fw: CTCACGTTTATTAAAAGAGCGTATTATTATGTTAGGTTCAC<br>Rv: GTGAACCTAACATAATAATACGCTCTTTTAATAAACGTGAG	pET301_SaClpP_D27E
pET301_SaclpP (SDM)	Fw: CTCACGTTTATTAAAAGCACGTATTATTATGTTAGGTTCAC<br>Rv: GTGAACCTAACATAATAATACGTGCTTTTAATAAACGTGAG	pET301_SaClpP_D27A
pET301_SaclpP (SDM)	Fw: CCTACAGTTATTGCAACAACAAACCGCGG<br>Rv: CCGCGGTTTGTTGTTGCAATAACTGTAGG	pET301_SaClpP_E9A
pET301_SaclpP (SDM)	Fw: GAACGTGCATATGATATATACTCAGCTTTATTAAAAGACC<br>Rv: GGTCTTTTAATAAAGCTGAGTATATATCATATGCACGTTC	pET301_SaClpP_R23A
pClpP11 (SDM)	Fw: GTGCGCAAGGTCAATCGACAGAAATTGAAATTG<br>Rv: CAATTTCAATTTCTGTCGATTGACCTTGCGCAC	pClpP11_A133S
pClpP11	Fw: GCG**CATATG**CAATTAATACCTACAGTC (*Nde*I)<br>Rv: GCG**CTCGAG**CTTTTTGTCTTCTGTGTG (*Xho*I)	pET22b_BsclpP-his
pClpP11_A133S	Fw: GCG**CATATG**CAATTAATACCTACAGTC (*Nde*I)<br>Rv: GCG**CTCGAG**CTTTTTGTCTTCTGTGTG (*Xho*I)	pET22b_BsclpP_<br>A133S-his
B. subtilis 168 gDNA (GA)	Fw: CTTTAAGAAGGAGATGGCCGATGCAATTAATACCTACAGT<br>CATTG<br>Rv: TCGAACTGCGGGTGGCTCCAAGCGCTCTTTTTGTCTTCTG<br>TGTGAGTC	pET11a_BsclpP-strep
+ pET11a-strep (GA)	Fw: AGCGCTTGGAGCCACCCGCAGTTC<br>Rv: CGGCCATCTCCTTCTTAAAG	
B. subtilis 168 gDNA (GA)	Fw: ACACATAAGGAGGAACTACTATGAATTTAATACCTACAGT<br>CATTG	pAPNC_Pspac_BsclpP
+ pAPNC (GA)	Rv: TTGGGCTAACGCCCGAATTCTTACTTTTTGTCTTCTGTGTG<br>Fw: GAATTCGGGCGTTAGCCC<br>Rv: AGTAGTTCCTCCTTATGTG	
B. subtilis gDNA (GA)	Fw: AGAAAGAGGAGAAATACTAGATGAATTTAATACCTACAGT<br>CATTG	pBS2E_BsclpP
+ pBS2E (GA)	Rv: CACTAGCACTATCAGCGTTATTACTTTTTGTCTTCTGTGTG<br>Fw: TAACGCTGATAGTGCTAG<br>Rv: CTAGTATTTCTCCTCTTTCTCTAGTATGTG	
pAPNC (GA)	Fw: TTCGCGGCCGCTTCTAGAGCCAAATAAAACGAAAGGCTC<br>Rv: CTGTAGGTATTAAATTCATCAGTAGTTCCTCCTTATGTG	pBS2E_Pspac_BsclpP
+ pBS2E_BsclpP (GA)	Fw: GATGAATTTAATACCTACAGTCATTGAACAAACGAAC<br>Rv: GCTCTAGAAGCGGCCGCG	

Sequencing oligonucleotides

Name	Oligonucleotide sequence (5´→3´)
Bs_clpP_seq_fw1	Fw: GCCAGCTTTTTGTTTGACCT
Bs_clpP_seq_fw2	Fw: GCAAT TGACATACGATGGTTTG
Bs_clpP_seq_rv1	Rv: CAGCTCTTGCAGGTTGTGTT
Bs_clpP_seq_rv2	Rv: GTGCTGATCTGGTTGTGAGGC
Bs_pAPNC_clpP_seq_fw	Fw: GTTGACTTTATCTACAAGGTG
Bs_pAPNC_clpP_seq_rv	Rv: GGCCCACTCAATGCCGTTAATAAT
Bs_pBS2E_clpP_seq_fw	Fw: CAATTAATGTGAGTTAGCTCAC
Bs_pBS2E_clpP_seq_rv	Rv: GAAGGTGAGCCAGTGTGACTC
Sa_clpP_flank_fw	Fw: GAAGGGAATTCAATAACCAAGTGATGCC
Sa_clpP_flank_rv	Rv: GGTGGTAACCCGGGTTCTGGTCAACAATGG
Sa_clpP_seq_fw1	Fw: AAGGAAAGAGAAAAGCGTCAAA
Sa_clpP_seq_fw2	Fw: AGACAAGCGTCAAACAATG
Sa_clpP_seq_rv1	Rv: TCATACAAAAAGAGCAGACAGCTT
Ef_clpP_seq_fw1	Fw: TGGTACACTATTTCTAAACGAAATCA
Ef_clpP_seq_rv1	Rv: AAAAAGCTTCCTTTGAAACAGA

Abbrevations: gDNA, genomic DNA; ORF BSU, open reading frame of *Bacillus subtilis* 168 (accession number NC_000964); C-terminal, carboxy-terminal; Bs, *Bacillus subtilis*; Sa, *Staphylococcus aureus*; Ef, *Enterococcus faecalis*; SDM, site-directed mutagenesis; restriction sites are indicated in underline and bold; GA, Gibson assembly

Table S2. Biochemical properties of ADEP-resistant *S. aureus* 133 ClpP mutants.

SaClpP mutant	Peptidase acitvity	Protease activity (with ADEP2)	SaClpXP activity	Oligomeric state
**WT**	**++**	**++**	**++**	**14-mer**
**Q132H**	**--**	**+**	**o**	**7/14-mer**
**A133T**	**--**	**-**	**--**	**7-mer**
**A133V**	**--**	**o**	**--**	**7-mer**
**N173D**	**--**	**-**	**--**	**7-mer**
**M190T**	**++**	**+**	**--**	**14-mer**

Respective activities are subdivided into five ranges from not active (**--**) to strongly active (**++**). Activites between 0-20 % of maximum activity are denoted with (**-**), intermediate acitivities from 20-80 % with (**o**), and strong activities between 80-100% with (**+**).

Table S3. Crystal data collection and refinement statistics

	***Sa*_ClpP**	***Sa*_ClpP:ADEP**
**Data collection**		
Space group	P1	$P6_522$
Cell constants		
a, *b*, *c* (Å)	95.2; 99.3; 107.5	96.5; 96.5; 586.8
α, β, γ (°)	105.2; 101.3; 117.7	90; 90; 120
Wavelength (Å)	1.0	1.0
Resolution range (Å)[a]	30-1.9 (2.0-1.9)	30-2.2 (2.3-2.2)
No. observations	414168	360540
No. unique reflections[b]	215061	80457
Completenes (%)[c]	90.3 (91.2)	95.6 (97.8)
R_{merge} (%)[a,d]	5.1 (54.0)	9.2 (59.2)
I/σ (I)[a]	9.0 (2.0)	9.6 (2.4)
**Refinement (REFMAC5)**		
Resolution range (Å)	30-1.9	30-2.2
No. reflections working set	204262	76395
No. reflections test set	10750	4021
No. non hydrogen	22698	10935
No. of ligand atoms	-	385
Solvent	1975	339
R_{work}/R_{free} (%)	17.3/21.7	20.4/23.8
R.m.s.d. bond (Å)/(°)[e]	0.009/1.23	0.008/1.25
Average B-factor ($Å^2$)	27.5	41.0
Ramachandran Plot (%)[f]	98.3 / 1.7 / 0	97.6 / 2.4 / 0
PDB accession code	6TTY	6TTZ

[a] The values in parenthesis for resolution range, completeness, R_{merge}, and I/σ (I) correspond to the highest resolution shell. [b] Data reduction was carried out with XDS and from a single crystal. Friedel pairs were treated as identical reflections. [c] $R_{merge}(I) = \Sigma_{hkl} \Sigma_j |[I(hkl)_j - \langle I(hkl)\rangle]| / \Sigma_{hkl} \Sigma_j I(hkl)_j$, where $I(hkl)_j$ is the jth measurement of the intensity of reflection hkl and $\langle I(hkl)\rangle$ is the average intensity. [d] $R = \Sigma_{hkl} | |F_{obs}| - |F_{calc}| | / \Sigma_{hkl} |F_{obs}|$, where R_{free} is calculated for a randomly chosen 5% of reflections, which were not used for structure refinement, and R_{work} is calculated for the remaining reflections. [e] Deviations from ideal bond lengths/angles. [f] Percentage of residues in favoured/allowed/outlier region.

III. Supporting References

[1] M. Gersch, K. Famulla, M. Dahmen, C. Göbl, I. Malik, K. Richter, V. S. Korotkov, P. Sass, H. Rübsamen-Schaeff, T. Madl, et al., *Nat. Commun.* **2015**, *6*, 6320.

[2] U. Gerth, J. Kirstein, J. Mostertz, M. Miethke, H. Kock, T. Waldminghaus, M. Hecker, *J. Bacteriol.* **2004**, *186*, 179–191.

[3] K. Katoh, D. M. Standley, *Mol. Biol. Evol.* **2013**, *30*, 772–80.

[4] C. Anagnostopoulos, J. Spizizen, *J. Bacteriol.* **1961**, *81*, 741–6.

[5] CLSI. *Methods for Dilution Antimicrobial Susceptibility Tests for Bacteria That Grow Aerobically; Approved Standard-Tenth Edition.* CLSI document M07-A10. Wayne, PA: Clinical and Laboratory Standards Institute; **2015**.

[6] E. S. Duthie, L. L. Lorenz, *J. Gen. Microbiol.* **1952**, *6*, 95–107.

[7] D. Frees, J. H. Andersen, L. Hemmingsen, K. Koskenniemi, K. T. Bæk, M. K. Muhammed, D. D. Gudeta, T. A. Nyman, A. Sukura, P. Varmanen, et al., *J. Proteome Res.* **2012**, *11*, 95–108.

[8] P. D. Fey, J. L. Endres, V. K. Yajjala, T. J. Widhelm, R. J. Boissy, J. L. Bose, K. W. Bayles, *MBio* **2013**, *4*, e00537-12.

[9] R. Novick, *Virology* **1967**, *33*, 155–166.

[10] D. Frees, S. N. A. Qazi, P. J. Hill, H. Ingmer, *Mol. Microbiol.* **2003**, *48*, 1565–1578.

[11] S. Gottesman, E. Roche, Y. Zhou, R. T. Sauer, *Genes Dev.* **1998**, *12*, 1338–1347.

[12] C. K. Smith, T. A. Baker, R. T. Sauer, *Proc. Natl. Acad. Sci. U. S. A.* **1999**, *96*, 6678–6682.

[13] M. Gersch, R. Kolb, F. Alte, M. Groll, S. A. Sieber, *J. Am. Chem. Soc.* **2014**, *136*, 1360–1366.

[14] K. Turgay, J. Hahn, J. Burghoorn, D. Dubnau, *EMBO J.* **1998**, *17*, 6730–6738.

[15] T. Morimoto, P. C. Loh, T. Hirai, K. Asai, K. Kobayashi, S. Moriya, N. Ogasawara, *Microbiology* **2002**, *148*, 3539–3552.

[16] N. Jahn, S. Brantl, H. Strahl, *Mol. Microbiol.* **2015**, *98*, 651–666.

[17] J. Radeck, K. Kraft, J. Bartels, T. Cikovic, F. Dürr, J. Emenegger, S. Kelterborn, C. Sauer, G. Fritz, S. Gebhard, et al., *J. Biol. Eng.* **2013**, *7*, 29.
